# Telling ecological networks apart by their structure: A computational challenge

**DOI:** 10.1371/journal.pcbi.1007076

**Published:** 2019-06-27

**Authors:** Matthew J. Michalska-Smith, Stefano Allesina

**Affiliations:** 1 Department of Ecology & Evolution, University of Chicago, Chicago, Illinois, United States of America; 2 Northwestern Institute on Complex Systems, Northwestern University, Evanston, Illinois, United States of America; Universitat zu Koln, GERMANY

## Abstract

Ecologists have been compiling ecological networks for over a century, detailing the interactions between species in a variety of ecosystems. To this end, they have built networks for mutualistic (e.g., pollination, seed dispersal) as well as antagonistic (e.g., herbivory, parasitism) interactions. The type of interaction being represented is believed to be reflected in the structure of the network, which would differ substantially between mutualistic and antagonistic networks. Here, we put this notion to the test by attempting to determine the type of interaction represented in a network based solely on its structure. We find that, although it is easy to separate different kinds of nonecological networks, ecological networks display much structural variation, making it difficult to distinguish between mutualistic and antagonistic interactions. We therefore frame the problem as a challenge for the community of scientists interested in computational biology and machine learning. We discuss the features a good solution to this problem should possess and the obstacles that need to be overcome to achieve this goal.

## Author summary

In the late 1960s, Mark Kac asked, "Can one hear the shape of a drum?" challenging readers to reconstruct the geometry of the drum from a provided list of overtones. Physicists and mathematicians found that, although in general, one cannot hear the shape of a drum, many of its properties, such as its area and perimeter, can be "heard". Here, we ask whether the type of interaction being represented in a network can be "seen" when inspecting its shape—for example, whether we can distinguish networks reporting interactions between plants and their pollinators (mutually beneficial) from those representing interactions between plants and their herbivores (beneficial only to the herbivores). We show that many types of nonbiological networks can be easily separated based on structural properties, whereas determining the type of interaction represented by an ecological networks is harder. We therefore turn this problem into a challenge for the scientific community. We argue that solving this problem would greatly benefit the field, and we discuss what the consequences of a failure might be.

## Introduction

Since the early days of the field, ecologists found themselves detailing the multitude of interactions occurring between different populations, such as predation, parasitism, and herbivory (all "antagonistic" interactions) or pollination, seed dispersal, and symbiosis ("mutualistic" interactions) [1, 2]. To make sense of these data, they built networks in which nodes are species and edges stand for interactions between species. Depending on the interaction being depicted by the edges, we speak of food webs (the edge *i* → *j* marks consumption of species *i* by *j*), host–parasite networks (*j* parasitizes *i*), pollination networks (animal *j* pollinates plant *i*), herbivory networks (animal *j* feeds on plant *i*), etc. Whereas the earliest published food web dates back more than a century [3], the past 20 years have seen a sharp increase in both the number of networks published and their quality (i.e., higher level of detail, larger number of species reported, and larger number of interactions, often including weighted edges).

Reducing a multidimensional object such as a network to a few numbers is a daunting task, but paralleling the progress of network analysis in other branches of science, ecologists set out to compute summary statistics on the empirical networks they collected; these metrics range from very simple measures, such as the size (number of nodes) and connectance (proportion of realized connections) of the network, to large-scale properties such as modularity (Are networks organized in blocks of dense connectivity loosely connected by few edges? [4]) and nestedness (Can interactions be organized as in a Russian doll—with specialist species choosing interactions among those of generalist species? [5–7]). As in other branches of science, ecologists also investigated degree distributions, motif profiles [8], k-cores [9], and many other network properties.

Here, we ask whether these metrics can be used to characterize the type of interaction being depicted by a network under the hypothesis that the type of interaction would affect network structure in a consistent and detectable way. Take a mutualistic network: both parties benefit from interacting, and they actively seek out this relationship from an evolutionary point of view—for example, plants reward pollinators with nectar, attract them with visual and olfactory cues, etc. Contrast this with herbivory, in which plants are desperate to avoid the interaction and display evolutionary strategies in the form of spines and thorns, toxic compounds, bark, etc. Given the contrasting forces at play, we might hypothesize that these processes give rise to networks with different structure [10, 11].

Previous analysis posited that mutualistic and antagonsitic networks’ properties would differ significantly (for example, mutualistic networks are believed to be "more nested" than antagonistic ones) [10–14]. However, this does not necessarily imply that network types can be separated based on these properties (even if the means of two distributions differ significantly, the overlap could be large enough to render a classification impossible). We frame the problem of detecting the type of interaction being represented in an ecological network by measuring its structure as a challenge to the community of scientists interested in networks, computational biology, and machine learning. We provide a database of networks of unprecedented size and completeness that can be used to test the quality of a solution, and we outline the principal hurdles that need to be overcome in order to solve this problem.

Before examining the details of the challenge, it is important to justify its timeliness. Classifying the type of an ecological network might seem like a pointless exercise: the ecologists that put it together knew perfectly well whether they were recording parasitism or pollination. However, solving this challenge is important for at least three reasons. First, showing that certain properties can be used to distinguish among networks is a post hoc justification for developing these metrics in the first place, thereby proving that they truly measure salient, relevant aspects of network structure. Second, a solution would provide a good justification for the representation of these data as networks—by compiling networks, we can infer properties of the system that would be difficult to assess otherwise. Third, with the advent of cheap, high-throughput molecular techniques, associations between species can be sampled at an unprecedented level of detail—for example, one can sequence the root of a tree to find all its fungal inhabitants, sequence insects to detect their endoparasites, etc. In this way, one could rapidly and reliably build networks of interactions between species—but what type of interactions would these species form? Some of the arbuscular mycorrhizal fungi sampled will be symbionts of the plant, whereas others will be parasites; in fact, the same species could act as either, depending on the ecological and environmental context. If we could find universal indicators of mutualistic versus antagonistic interactions, then we could make an educated guess on the type of interaction represented in these networks by just measuring a few of their properties. Note that, to keep the task as simple as possible, here we ask the broad question, What type of interaction is represented in this network? However, the same question could be asked of each single edge, as ecological networks are typically composed of a variety of interactions [27, 30].

To illustrate the details of the computational challenge with a simple, solvable example, we draw upon a large data set of nonecological networks we have compiled for this purpose. We show that it is quite easy to separate these classes of networks, even using elementary methods. We then turn to ecological networks, showing that the task is much more difficult: not only can we not separate cleanly mutualistic from antagonistic ecological networks, but we cannot even separate ecological networks from the remaining nonecological networks.

## Materials and methods

We built undirected, unweighted bipartite networks recording the connections between actors and movies (“actor collaboration”), authors of scientific articles and the journals in which they have published (“authorship”), lawmakers votes on laws (“legislature”), microbial organisms and parts of the body where they are found (“microbiome”), and city neighborhoods and crime occurrences (“crimes”). These systems were chosen based on two characteristics: (1) for each category, multiple (100 or more) networks could be built using the same methodology and (2) the networks within each category would display a large range of sizes and levels of connectivity.

We also amassed a large database of more than 500 bipartite ecological networks, representing either antagonistic (host–parasite, host–parasitoid, bacteria–phage, plant–herbivore) or mutualistic (plant–pollinator, plant–seed disperser, ant–plant, anemone–fish) interactions.

The summary statistics on the size and connectivity for each class of networks are reported in Table 1; the details of how the networks were constructed and the references for the published networks are provided in the Supporting information.

**Table 1:** Summary statistics on the size and fill of collected networks.

Type	Metric	Minimum	Maximum	Mean	Median
Actor Collaboration (155)	Connectance	0.0002	0.072	0.005	0.0026
	Links	258	78,145	12,658.652	6,753
	Rows	15	6,675	1,116.394	568
	Columns	239	43,872	7,808.723	4,832
Antagonism (197)	Connectance	0.0078	0.9301	0.27	0.2361
	Links	14	5,203	165.472	82
	Rows	5	749	38.67	18
	Columns	5	888	34.274	21
Authorship (109)	Connectance	0.0303	0.0588	0.04	0.0388
	Links	2,186	16,611	9,479.532	9,939
	Rows	86	712	389.349	396
	Columns	438	681	623.138	639
Crimes (1,816)	Connectance	0.0457	0.6053	0.25	0.2406
	Links	12	470	153.214	120
	Rows	7	77	39.834	33
	Columns	5	64	22.273	21
Legislature (245)	Connectance	0.1715	0.8455	0.593	0.6056
	Links	277	367,956	19,162.89	7,092
	Rows	16	972	167.898	138
	Columns	10	1,427	151.698	87
Microbiome (203)	Connectance	0.0621	0.1891	0.103	0.0982
	Links	7,585	46,936	19,396.808	18,500
	Rows	4,559	16,163	8,625.236	8,442
	Columns	9	44	22.616	23
Mutualism (360)	Connectance	0.0173	0.6875	0.202	0.1907
	Links	12	15,255	183.733	58
	Rows	5	1,044	47.244	27
	Columns	5	647	27.294	15

For references and a detailed description, see Supporting information.

### Choice of metrics

Given the great interest in network analysis, there are hundreds of network metrics one could use to attempt to characterize the different classes of networks. When it comes to ecological networks, however, two of their most accessible properties, the size (number of nodes) and connectance (proportion of realized links), should not be used for this purpose. In fact, an ecological network’s size and connectance mostly reflect the experimental design and sampling effort rather than structural differences from other networks. These networks typically integrate information through space (e.g., Where does my ecosystem "end"?) and time (Shall we build a network for each day of sampling? Week? Year?)—sampling more extensively in time and space will surely result in a larger number of species and connections.

Given these limitations, one should choose metrics that either are not influenced by size and connectance or can be rescaled to remove their effects. For the illustrative example we use to detail the computational challenge, we are going to follow the latter approach, though the former would be superior, if we were to find network properties that are insensitive to size and connectivity.

For each network, we measure the two largest eigenvalues of the adjacency matrix, which are associated with important network properties: it is well known that the first eigenvalue (spectral radius) of the adjacency matrix is maximized in perfectly nested networks [15], whereas the second eigenvalue will separate from the bulk of the spectrum in strongly modular networks [16]. Because both eigenvalues are expected to grow whenever we add nodes and connections, we normalize them by computing their expectation under two null models: an Erdős–Rényi, random bipartite graph (in which the number of nodes and connections are preserved, but nodes are connected at random, [17]), and a configuration model (in which the nodes’ degrees are preserved, but again, wiring is random [18, 19]). By computing the relative error we make when using these expectations rather than the observed values, we attempt to remove the trivial effect of size and connectance (Table 2).

**Table 2:** Metrics used in a PCA.

Metric	Formula	PC1	PC2	PC3
Configuration model *λ*_1_ relative error	$1-\lambda_{1}^{cm}/\lambda_{1}$	0.578	−0.576	0.578
Erdős–Rényi *λ*_1_ relative error	$1-\lambda_{1}^{er}/\lambda_{1}$	0.646	−0.111	−0.755
Marchenko–Pastur *λ*_2_ relative error	$1-\lambda_{2}^{mp}/\lambda_{2}$	0.499	0.810	0.308
**Percent Explained Variance**:		**53.944**	**27.659**	**18.397**

PCA run on the correlation of 500 nonecological bipartite networks with each of the first three principal component vectors. Here, *λ* is an eigenvalue of the adjacency matrix of the network, and the subscript indicates its position when sorted in descending order; the superscript indicates that the value is an approximation of the eigenvalue under a given randomization protocol (Supporting information). Abbreviations: PC1, principal component 1; PC2, principal component 2; PC3, principal component 3; PCA, principal component analysis

### Mapping networks

Several methods of classifying objects have been developed in the literature on statistics and machine learning, and picking a single method from such an embarrassment of riches is hard. To keep the illustration of the challenge as simple as possible, here we measure the three spectral quantities introduced previously, thereby representing each network as a single point in a three-dimensional space. To better visualize the position of the networks in this space, we perform a principal component analysis (PCA), projecting the network positions on the plane defined by the first two components. Metaphorically, we are mapping out the "network space" such that, if we were to have chosen meaningful quantities, two networks belonging to the same class should be close, and the various network classes should form clusters that are well separated.

The use of a PCA facilitates the illustration of the three properties we believe any good solution to this challenge should possess:

#### Generality

Having trained on a specific data set, the method should be capable of classifying new networks without the need of repeating the calculation.

To illustrate this point, we defined the principal components using a set of 100 networks of each kind among the nonecological sets and projected them on the space defined by the first two components (Fig. A in S1 Supporting Information, left pane). The networks cluster by type, and the two axes explain much of the variation (Table 2). When we project onto this space all the remaining nonecological networks, we find that, despite being "out of fit", the new networks fall exactly where they should be: the method can be used to classify new networks based on their position in the plane.

#### Specificity

The method should not be able to classify the networks when the structure has been sufficiently perturbed.

In particular, if we were to randomize the networks, they should all be classified as "random networks" rather than retaining their identity: at its most basic level, specificity is needed to prove that the analysis is not overly influenced by size and connectivity.

The top panel shows the empirical, nonecological networks, a subset of which were used to construct the principal component space—which is maintained throughout the remaining plots. The middle panel keeps the same axes, but instead of projecting the empirical networks, it uses the properties measured for their randomized version under the Erdős–Rényi model. Likewise, the bottom panel shows a point for each network once randomized using a configuration model; this randomization is more computationally taxing; hence, there are fewer data points in this panel, especially for the large and sparse actor collaboration networks (Supporting information).

**Fig 1:**
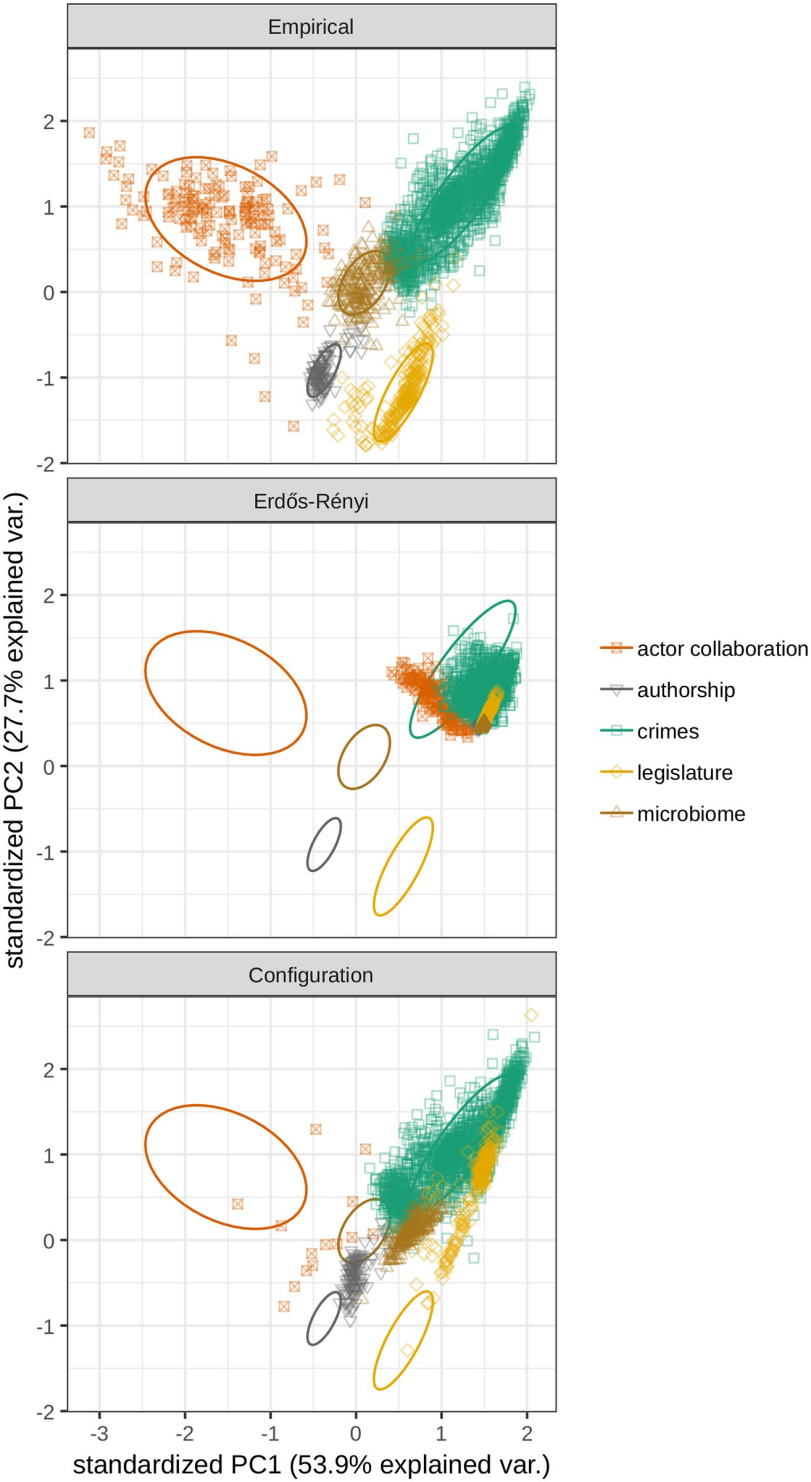
PCA biplots of the two most explanatory principal components. Each point indicates a single network, and the ellipses are drawn to contain approximately 68% of the points in each network type, i.e., one standard deviation if the points were to follow a bivariate normal distribution. PC1, principal component 1; PC2, principal component 2; PCA, principal component analysis; var., variation.

To illustrate this point, we produced two randomized versions of each original network: an Erdős–Rényi random bipartite graph [17] with the same number of nodes in each class and the same number of connections as in the original graph and a network built with a configuration model [18, 19], preserving also the degree sequence of the original graph. When we compute the three metrics illustrated above for each randomization and project the resulting values on the PCA space, we find that almost all networks cluster together under the Erdős–Rényi model (Fig 1); note that ideally these should collapse to a point—the remaining scatter indicates that our approximation is not accurate for very sparse or very small networks, possibly because the size of the fluctuations of these quantities around their mean is influenced by size and connectance. When we analyze the data from the configuration model, we start seeing a clear separation of the clusters, meaning that degree distributions differ significantly between classes. In the same spirit, one could devise other randomizations preserving or foregoing certain properties, thereby probing whether they can be used to distinguish networks.

#### Scalability

There should be different levels of classification, displaying a hierarchical structure.

Previously, we stated that the method should be able to consistently distinguish networks of different classes—this language is intentionally imprecise. We use "class" loosely such that it could refer to separating networks representing mutualistic interactions from those depicting antagonisms, distinguishing hummingbird pollination networks from those in which the pollinators are bees, or even just recognizing ecological networks. This ambiguity is intentional in order to remain agnostic with respect to what the appropriate level of differentiation should be or even whether there should be one primary level. Analogous to the issue of classifying organisms in a taxonomy, there should be a hierarchy of differentiation that can be used to infer relevant aspects of these systems. For this reason, a successful method should be able to identify levels of segregation within the data according to some hierarchy of network similarity.

**Fig 2:**
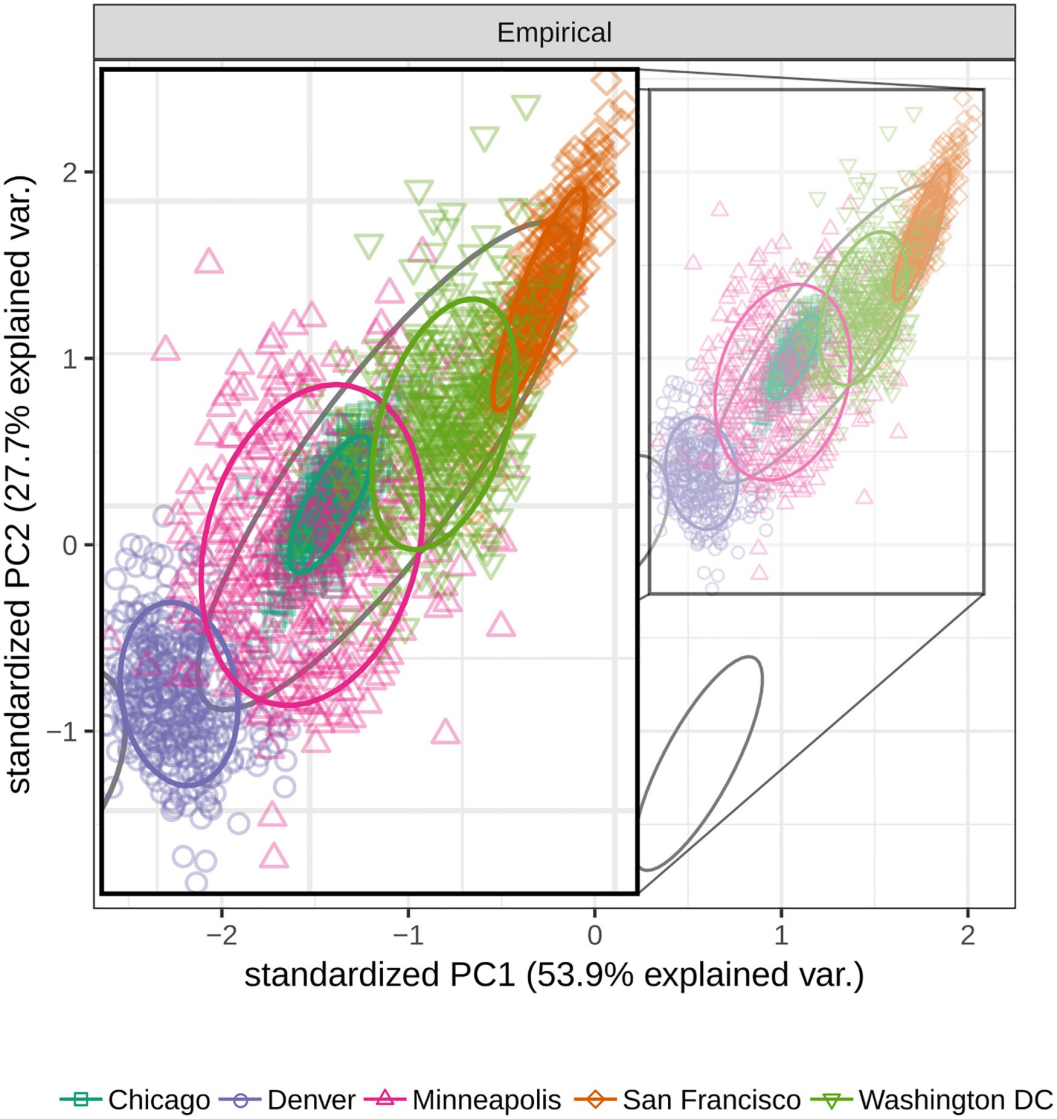
Subsetting the points from the top panel of Fig 1 to include only the networks of crime locations, we now color the points according to the city from which each crime network was collected. Note that, though all crime networks cluster together in Fig 1, those from the same city cluster more tightly, albeit with significant overlap between cities. This indicates that there is additional structure beyond that used to disambiguate crime networks from those of authorship, legislature, etc., and by looking at these finer differences, more subtle distinctions can be made. PC1, principal component 1; PC2, principal component 2; var., variation.

We demonstrate this property in Fig 2, in which we look more closely at the cluster of crime networks in the top panel of Fig 1. Although Fig 1 shows that crime networks cluster more closely to one another than to networks of actor collaboration, legislature voting records, or human microbiome networks, Fig 2 reveals substructure within this cloud of points corresponding to networks collected for the same city: although all crime networks cluster together, those in the same city cluster more tightly.

## Results and discussion

### Adding ecological networks to the map

Having demonstrated some level of success in achieving generality, specificity, and scalability, we now turn to the focus of our challenge: applying the method to ecological interaction networks.

**Fig 3:**
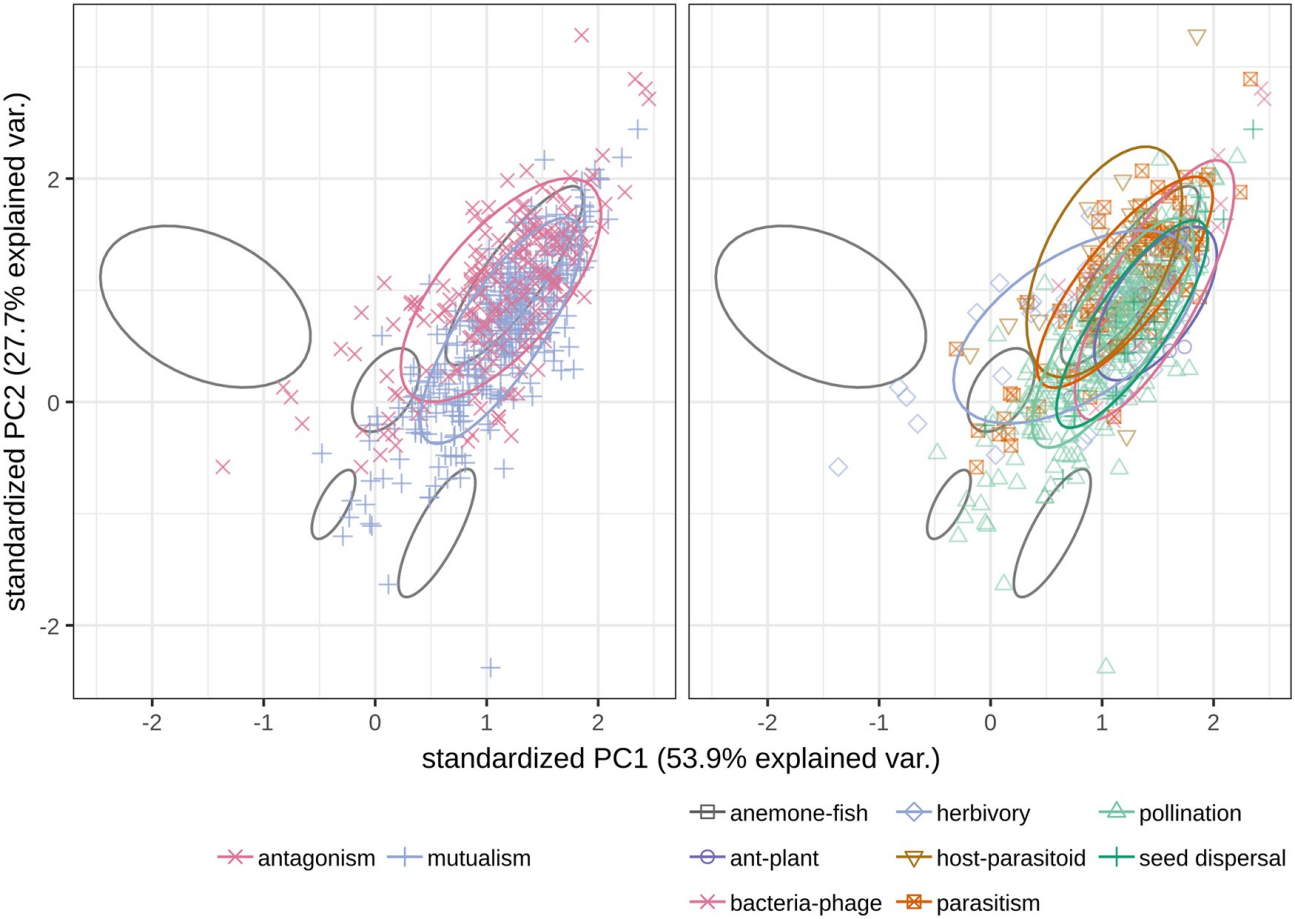
Left: overlaying more than 500 ecological networks representing either mutualistic or antagonistic interactions, we find that they do not cluster as nicely as the nonecological ones did, suggesting that there is much more variation within ecological network structure than between the classes of nonecological networks examined here. Right: this result holds when the networks are instead labeled according to the specific type of mutualism/antagonism they describe. PC1, principal component 1; PC2, principal component 2; var., variation.

When we project our ecological networks onto the principal component space constructed above, we find that they span a large area of the map, overlapping many of the categories that were previously well separated. Moreover, antagonsitic and mutualistic networks overlap substantially (Fig 3). These results persist when only highly replicated networks are used, when ecological networks are utilized in the construction of the principal component space, and when alternative metrics are used to construct the PCA (Supporting information).

**Fig 4:**
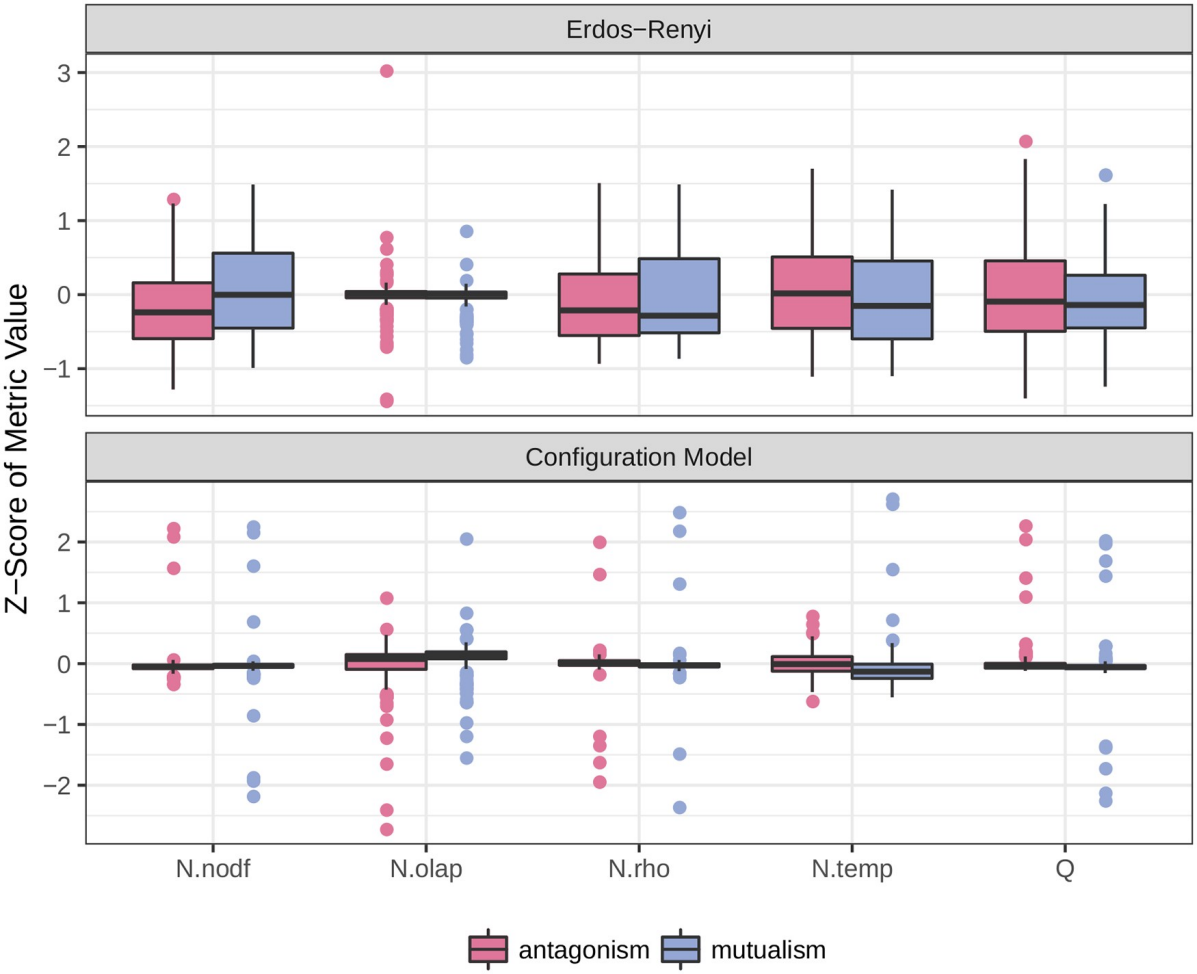
We compared networks of ecological interactions based on their value of nestedness and modularity. We randomized each empirical web 1,000 times according to an Erdős–Rényi (top) or configuration model (bottom), each modified slightly to produce only connected graphs (Supporting information). For each of these randomizations, we calculated nestedness [in a variety of ways, including NODF (N.nodf; [20]), overlap (N.olap; [21]), spectral radius (N.rho; [15]), and temperature (N.temp; [5])] and modularity (*Q*; [4]). We plot here the Z-score of the empirical value for each measure with respect to the 1,000 randomizations. For the Erdős–Rényi randomizations, the only significant (using Welch’s [22] *t* test; Supporting information) difference is for NODF, showing mutualistic networks to be more nested than antagonistic ones. For the configuration null model, only the Overlap measure of nestedness shows significant differences (Supporting information), but in this case, networks of antagonistic interactions are deemed more nested than those depicting mutualisms. Modularity does not vary significantly between the two types of ecological interaction networks.

The results presented in Fig 3 are somewhat surprising—Why are ecological networks hard to classify when there have been previous publications claiming success? Using our new, larger data set, we can reassess the proposed differences between networks of antagonistic and mutualistic interactions. Using a smaller data set, Thébault and Fontaine [12] had found significant differences in nestedess [20] and modularity [4] between mutualistic and antagonistic networks. We repeat and extend this analysis by looking at additional measures of nestedness, constraining the null model to ensure connectedness, and adding a second null model (Fig 4 and Supporting information). We find conflicting support for the use of these measures in the classification of ecological networks. Indeed, both the size and direction of these differences can be influenced by the choice of metric. Moreover, even in the case of a significant difference, there is substantial overlap in the metric distributions, suggesting that these measures cannot alone reliably distinguish network types (i.e., though the means could be significantly different from a statistical standpoint, the high variance would render a classification based on these features difficult).

### Choice of method and metrics

For each potential method to solve the problem outlined here, one of the key decisions is which (and how many) metrics to include in the analysis, and a plethora of statistics are currently available. For illustrative purposes, we kept the number of metrics to a minimum, but in the Supporting information, we experiment with many more, obtaining the same qualitative results.

Note that the choice of metrics is not inconsequential: including too few will provide insufficient power to distinguish between similar network types, whereas too many can lead (in some modeling frameworks) to noise or multicollinearity that can obscure meaningful differences. There are also logistical concerns, as some metrics require much more computation time than others. Certain estimates of modularity, for instance, require first identifying the appropriate clustering of the species involved—a computationally intensive problem [23].

Similarly, there are a variety of methods that could be applied to this problem. Although we used simple spectral measures and PCA to render the network space in two dimensions here, a number of techniques, in particular those in the rapidly growing field of machine learning, are arguably better suited to this task. However, although popular machine learning techniques typically require the availability of a properly labeled "training set", here we achieved a good separation between different classes of nonecological networks without supplying any label—we simply mapped out the networks based on their spectral properties.

We used PCA to produce a two-dimensional "map" onto which we can place new networks. Yet PCA has a number of shortcomings. Foremost is that the space constructed varies with the data used. If we were to include more, or different, nonecological networks in the generation phase, we would obtain a different map. Additionally, we have not run statistics on the clustering we observe in principal component space, introducing an element of subjectivity into the analysis as stated. Alternative or additional methods could be implemented that incorporate objective grouping to enhance the robustness of any results.

Finally, we have analyzed unweighted, undirected graphs, despite the fact that many modern ecological networks include measures of interaction strengths [24, 25]. Although weights are likely to help with separating different classes of networks, many of the most popular network properties (e.g., motif profiles) are difficult to extend to weighted networks.

## Conclusion

In 1966, Mark Kac asked, "Can One Hear the Shape of a Drum?" Is it possible to infer the shape of a drumhead from a list of the overtones it produces [26]? Although the short answer is no, many important properties of the drum, such as its area and perimeter, can be "heard" distinctly.

Here, we asked whether the type of interaction being represented in a network can be "seen" from its structure. We have shown that the classification is easy for several classes of nonecological networks: even foregoing size and fill, the structure of these networks is distinctive enough to allow for a reliable classification.

Yet the same task is more complicated when examining ecological networks. In this case, the naïve approach that successfully classified nonecological networks fails completely, and adding more metrics or using different approaches seems not to help with the classification (Supporting information). Because of this fact, we framed the problem as a computational challenge to the community of scientists interested in networks and machine learning. We have compiled a large data set that can be used to explore and validate potential solutions, and we have detailed three important properties that need to be fulfilled.

Succeeding in this challenge would have profound implications for the field: although networks are surely convenient objects for storing information about species’ interactions, a positive solution would prove that, by representing ecological communities as networks, we gain insights that would be precluded otherwise. Moreover, as for other areas of machine learning (e.g., face recognition), with the advent of new techniques for the high-throughput production of networks, this question could rapidly move from the purely academic to the applied side. Although current approaches to constructing networks are often laborious, we know what these networks represent. In the future, our ability to produce networks could surpass our ability to identify what interactions represent, leading to novel confusions and challenges. Finally, network metrics that can capture essential aspects of the ecology of these systems would surely deserve a special place in the hearts and minds of ecologists.

But what if a solution to this challenge cannot be found? This failure could be due to a number of reasons. First, unweighted, bipartite graphs representing a single type of interaction might not carry sufficient information for this task. Many of the ecological networks currently being published quantify interactions (e.g., measuring the number of visits a pollinator pays to a plant), and a few started detailing the different types of interactions between the species using multidimensional networks [27–30]. As such, the solution could present itself once one were to extend the appropriate metrics to weighted, multidimensional networks. Second, it could be that ecological networks have greater within-class variation than the other classes examined here. Third, the difference between mutualistic and antagonistic relationships might not be the main driver of network structure, and other, more-meaningful classifications could emerge that would be easy to infer when examining ecological network structure. Ultimately, it is likely a combination of these explanations and others not listed here, the complete description of which remains a topic for future investigation. In any case, there would be much to learn from a failure, and to celebrate in case of success.

## Supporting information

S1 Supporting Information Data collection sources and procedures, detailed and additional methods, and supplementary figures and tables. (PDF)
