# Supplementary material for: Telling ecological networks apart by their structure: A computational challenge
Source: PLoS Comput Biol. 2019 Jun 27;15(6):e1007076. doi: 10.1371/journal.pcbi.1007076 (PMC6597030; doi:10.1371/journal.pcbi.1007076)
Supplement: S1 Supporting Information — (PDF) [file pcbi.1007076.s001.pdf]

# Telling ecological networks apart by their structure: a computational challenge
# Supplementary Information

Matthew J. Michalska-Smith & Stefano Allesina

May 13, 2019

## Contents

**S1 Network Data** **2**
S1.1 Crime networks . . . 2
S1.2 Legislature networks . . . 2
S1.3 Microbiome networks . . . 2
S1.4 Authorship networks . . . 2
S1.5 Movie-Actor networks . . . 3
S1.6 Ecological interaction networks . . . 3
S1.7 Censoring networks . . . 4

**S2 Randomizations** **4**

**S3 Matrix representation of bipartite networks and their spectra** **4**

**S4 Metrics Collected** **5**
S4.1 Spectral properties . . . 5
S4.2 Expectation for the largest eigenvalues of the randomized networks . . . 5
S4.3 Other network properties . . . 6

**S5 A Note on Methodology** **8**

**S6 Uninformative Variation** **9**

**S7 Alternative Metrics** **11**

**S8 Focusing on Nestedness and Modularity** **15**

**S9 Alternative Ecological Data** **17**
S9.1 More Dimensions . . . 19

**S10 Including Ecological Data in the Fitting** **22**

**S11 Machine Learning Approaches** **28**
S11.1 Using same metrics as main text . . . 28
S11.2 Using the full set of metrics . . . 29
S11.3 Directly Parsing Ecological Networks . . . 30
S11.4 Directly Parsing Ecological Networks—Subcategorizations . . . 31

**S12 Code availability** **32**

# S1 Network Data

In this section, we briefly describe how the ecological and non-ecological networks were collected. All the networks have been stored in a Git repository (`http://git.io/f4y4o`), with each file reporting the details on how the network was collected, as well as the reference to where it was published (if relevant).

## S1.1 Crime networks

The networks represent daily crime occurrence by neighborhood. Nodes stand for either a crime or a city neighborhood, and edges indicate which crimes took place in which neighborhood on a given day of the year. We compiled a network for each day of 2016 and for several cities: Chicago, Denver, Minneapolis, San Francisco, and Washington DC. For each network, we took the largest connected component (in case of disconnected networks).

## S1.2 Legislature networks

Annual networks of legislature voting rolls. Nodes indicate either a legislator or a bill and links indicate positive votes of a given legislator for a given bill. Data was collated for the United States (House and Senate), the United Nations General Assembly, and the European Parliament. For each network originally containing all bills voted on in a given year and all legislators who voted on at least one of those bills, the largest connected component (in case of disconnected networks) was extracted.

## S1.3 Microbiome networks

In our individual microbiome networks, nodes are either location on the body of an individual or operational taxonomic units (OTUs) of bacteria found at the body location. Edges mark the presence of a given OTU at a particular bodily site. We used OTUs based on the 16S rRNA variable region 3-5 (V35), which was analyzed for all the samples (about 5000) by the NIH Human Microbiome Project. For each of the subjects, samples were collected for 15–18 body sites. We built a network for each individual associated with more than 10 samples. In case of multiple visits by the same subject, the same body sites could have been sampled more than once, in which case we kept the samples as distinct nodes. As such, the number of nodes representing sites can be larger than 18. To build the networks, we downloaded the data on the samples and connected it with the corresponding OTU table.

## S1.4 Authorship networks

These networks connect authors to journals where they have published across their career. For example, the network ”American Naturalist 2016” takes all the authors who have published in The American Naturalist in 2016, and connects them with the journals where they have published throughout their career.

We downloaded from Scopus all the abstracts published in 10 ecological journals (*The American Naturalist*, *Ecography*, *Ecological Applications*, *Ecological Monographs*, *Ecology*, *Ecology Letters*, *Journal of Animal Ecology*, *Journal of Applied Ecology*, *Journal of Ecology*, and *Oikos*) for the years 2006 to 2016. We then extracted all authors ID found in the metatadata associated with each abstract ($31,308$ unique authors were represented). For each author, we collected from Scopus the names of the journals in which they had published their research. We retained all journals in which at least 100 of the authors had published (687 journals). Finally, we constructed the network connecting authors to journals and took the giant component, comprising $20,087$ authors and 687 journals. Each of the networks analyzed in this work is obtained subsetting this giant component, by selecting only the authors who have published in a given journal/year combination (*e.g.*, *The American Naturalist*, 2016).

## S1.5 Movie-Actor networks

These networks connect actors with the movies in which they acted. For each of the $201,346$ movies listed in the Movie Data base, we have downloaded the list of actors in the credits, as well as the production year and the genre(s) (up to four genres were recorded). We then built a network for each genre and period (spanning a few years, for example "Action 1996-2000" or "Crime 1900-1950"; the different periods were chosen to avoid networks that were to small or too large). For each network, we retained only the giant component.

## S1.6 Ecological interaction networks

These networks detail species interactions among two sets of species. They can be further subdivided into separate sub-types, reported below. In all cases, both classes of nodes represent species (or other taxonomic units, especially in the case of bacteria and viruses), and edges connecting the nodes stand for species' interactions (*e.g.*, pollination, parasitism). Because these networks have been taken from the ecological literature, we simply report the references to the original studies, containing a detailed description of how the data were collected.

### S1.6.1 Antagonism

- Host-Parasite (109) [Hadfield et al., 2013, Arai and Mudry, 1983, Arthur et al., 1976, Bangham, 1955, Bellay et al., 2013, Dechtiar, 1972, Krasnov et al., 2012, Leong and Holmest, 1981, Pilosof et al., 2013]
- Plant-Herbivore (41) [Basset et al., 1996, Blüthgen et al., 2006, Bodner, 2009, Coley et al., 2006, Ibanez et al., 2013, Janzen et al., 1980, 2003, Joern, 1979, Leather, 1991, Lewis et al., 2002, Loye and Zuk, 1992, Macfadyen et al., 2009, Nakagawa et al., 2015, Novotny et al., 2005, 2012, Prado and Lewinsohn, 2004, Starỳ et al., 2008, Tavakilian et al., 1997, Ueckert et al., 1976]
- Bacteria-Phage (27) [Barrangou et al., 2002, Capparelli et al., 2010, Comeau et al., 2005, 2006, Doi et al., 2003, Flores et al., 2011, Hansen et al., 2007, Holmfeldt et al., 2007, Kankila and Lindström, 1994, Krylov et al., 2006, Langley et al., 2003, Middelboe et al., 2009, Miklič and Rogelj, 2003, Poullain et al., 2008, Quiberoni et al., 2003, Rybniker et al., 2006, Seed and Dennis, 2005, Stenholm et al., 2008, Sullivan et al., 2003, Synnott et al., 2009, Wichels et al., 1998, Zinno et al., 2010]
- Host-Parasitoid (23) [Barbosa et al., 2003, Carvalheiro et al., 2008, Le Corff et al., 2000, Macfadyen et al., 2009, Stireman and Singer, 2003, Sugiura, 2007]

### S1.6.2 Mutualism

- Plant-Pollinator (217) [Brosi et al., 2017a,b, Carstensen et al., 2018, 2017, Kaiser-Bunbury et al., 2010, 2014, Magrach et al., 2018, 2017, Arroyo et al., 1982, Barrett and Helenurm, 1987, Bartomeus et al., 2008, Bezerra et al., 2009, Bundgaard, 2003, Burkle et al., 2013, Clements and Long, 1923, Dicks et al., 2002, Dupont et al., 2003, Dupont and Olesen, 2009, Elberling and Olesen, 1999, Herrera, 1988, Hocking, 1968, Inoue, 1990, Inouye and Pyke, 1988, Kaiser-Bunbury et al., 2014, Kakutani et al., 1990, Kato et al., 1990, 1993, Kato, 1996, 2000, Kevan, 1970, Lundgren and Olesen, 2005, McMullen, 1993, Medan et al., 2002, Memmott, 1999, Montero, 2005, Mosquin and Martin, 1967, Motten, 1982, Olesen et al., 2002, Ollerton et al., 2003, Percival, 1974, Petanidou, 1991, Primack, 1983, Ramirez, 1989, Ramirez and Brito, 1992, Robertson, 1929, de Mendonça Santos et al., 2010, Schemske et al., 1978, Small, 1976, Smith-Ramírez et al., 2005, Stald, 2003, Tur et al., 2013, Vázquez, 2002, Weiner et al., 2011, Yamazaki and Kato, 2003, Gilarranz et al., 2015, 2014, Orford et al., 2016b,a, Lara-Romero et al., 2016b,a, Trøjelsgaard et al., 2015, Trøjelsgaard et al., 2015]
- Plant-Seed disperser (41) [Baird, 1980, Beehler, 1983, Carlo et al., 2003, Crome, 1975, Frost, 1980, Galletti and Pizo, 1996, Guitian Rivera and Callejo Rey, 1983, Hamann and Curio, 1999, Jordano, 1985, Lambert, 1989, Mack and Wright, 1996, NOMA, 1997, Poulin et al., 1999, Schleuning et al.,

2011, Silva, 2002, Snow and Snow, 1971, 1988, Sorensen, 1981, Tutin et al., 1997, Wheelwright et al., 1984]

- Ant-Plant (11) [Blüthgen et al., 2004, Davidson et al., 1989, Fonseca and Ganade, 1996, Passmore et al., 2012]
- Anemone-Fish (1) [Ollerton et al., 2007]

### S1.7 Censoring networks

For all figures in the main text and in this supplement, any networks collected which had fewer than five nodes in either category (*e.g.*, plants or pollinators, crimes or neighborhoods) were excluded from further analysis and summary tables. In general, we believe that the type of analysis described in this work is only feasible for networks that a) are sufficiently large (because fluctuations in network metrics are going to overwhelm the "signal" in small networks); and b) sufficiently connected (if a network is close to a tree, or another barely connected graph, there is no structure to investigate; the same holds for networks where almost all edges are realized).

## S2 Randomizations

For each network we collected, we created two randomized versions. The first randomization produces a network containing exactly the same number of nodes and links, but having the links rearranged such that any two nodes have the same probability of being connected. This randomization yields an Erdős-Rényi random bipartite graph with the same size and fill as the original. Because the original networks are surely connected (for each network, we only kept the giant connected component), we repeatedly randomized each network until we obtained a connected graph. For sufficiently high connectance, the probability of obtaining a connected graph is close to one, and as such the computation is quick. For sufficiently low connectance, however, the probability of obtaining a connected graph rapidly approaches zero [Staniczenko et al., 2013], such that this naive method for randomizing the network would require an unreasonably long time. In these cases, we modified the randomization scheme by first drawing a random spanning tree through the nodes, and then randomly assigning the remaining links (to bring the total number up to that of the original network) as described above. This should not bias our randomizations, as a spanning tree is a necessary backbone of any connected network.

The second randomization produces a random bipartite graph with the same degree sequence as the original (*i.e.*, each **node** maintains the same number of connections as it had in the original network). This is known as a "configuration model". For this randomization, we utilized a fast, unbiased routine called the "curveball algoritm" [Strona et al., 2014]. In this case, it is more compuationally taxing to ensure connectedness of the randomized network, and therefore we discarded disconnected graphs and re-randomized from the original network again until a connected graph was identified. For several networks (especially the actor-movie set), despite more than 1000 attempts, we were still unable to find a connected network. This is often due to very low connectance, which reduces the number of possible connected networks with the desired degree distribution. For these cases, we omitted those networks from configuration model panels in the relevant figures. Due to the unbiased nature of the curveball algorithm, this process of discarding and re-randomizing is not expected to influence our analysis. Importantly, the curveball algorithm only produces simple graphs (*i.e.*graphs with neither links connecting a node to itself (self-links) nor multiple links connecting the same pair of nodes (hyperedges)). Since all of our empirical networks are simple graphs, this constraint increases the relevance of the network-structure-space we are sampling for comparison.

## S3 Matrix representation of bipartite networks and their spectra

Any bipartite network can be represented as a $n \times m$ incidence matrix $\mathbf{B}$, where $n$ is the number of rows (nodes of the first class), and $m$ the number of columns (nodes of the second class), and $B_{ij} = 1$ if node $i$

and $j$ are connected, and 0 otherwise. Without loss of generality, we assume $n \geq m$ in what follows. The graph can be also represented as a symmetric adjacency matrix:

$$\mathbf{A} = \begin{pmatrix} \mathbf{0} & \mathbf{B} \\ \mathbf{B}^T & \mathbf{0} \end{pmatrix} .$$

Where a superscript $T$ signifies the transpose of the associated matrix. The spectrum (*i.e.*, the set of eigenvalues) of the adjacency matrix of a bipartite network is composed of real eigenvalues and symmetric about 0: if $\lambda$ is an eigenvalue of $\mathbf{A}$, then $-\lambda$ is also an eigenvalue. Up to $2m$ eigenvalues can be different from zero, while the remaining eigenvalues are all 0. This means that in general the adjacency matrix will be rank-deficient whenever $n \neq m$. The singular values of the bipartite network, $\sigma_1, \sigma_2, \ldots \sigma_m$ are the eigenvalues of $\mathbf{B}^T\mathbf{B}$. The eigenvalues of $\mathbf{B}\mathbf{B}^T$ are the same of those of $\mathbf{B}^T\mathbf{B}$, with the addition of $n-m$ zero eigenvalues. The eigenvalues of $\mathbf{A}$ are the square roots of the singular values.

# S4 Metrics Collected

For each network, including the two randomized versions of each empirical network, we measured a number of structural properties. These included network size (number of rows and columns in the matrix representation of the bipartite network) and fill (absolute number of links as well as the ratio of the number of links to the number of possible links, termed "connectance"), and a number of other properties.

In this section, we list the metrics we collected, along with a brief explanation or references to the literature. We describe in detail how the metrics used in the main text for our example were obtained.

## S4.1 Spectral properties

For each network, we recorded the three "rightmost" (largest positive) eigenvalues of the adjacency matrix, $\lambda_1$, $\lambda_2$ and $\lambda_3$ as well as the three largest eigenvalues of the Laplacian matrix $\mathbf{L} = \mathbf{D} - \mathbf{A}$, where $\mathbf{D}$ is a diagonal matrix whose coefficients report the degrees of each node. We also measured the inverse participation ratio for the three leading eigenvectors of the adjacency matrix, quantifying localization [Pastor-Satorras and Castellano, 2016]: if $w$ is a unitary eigenvector of $\mathbf{A}$, $IPR = \sum_i w_i^4$. Finally, we measured the algebraic connectivity of the network, taken as the second smallest eigenvalue of the Laplacian matrix [Fiedler, 1973].

## S4.2 Expectation for the largest eigenvalues of the randomized networks

For each network, we want to also record the expected values of $\lambda_1$ and $\lambda_2$, which we will then use to remove the dependency on size and fill. These approximations are based on the assumption that we are estimating the eigenvalues of a random graph, either with a specified degree sequence (configuration model; only the spectral abscissa ($\lambda_1$)), or with a given size and fill (Erdős-Rényi for the spectral abscissa and Marchenko-Pastur for the second largest eigenvalue). We omit the calculations here for the expected value of the second largest eigenvalue of a configuration model random graph, as we found that, while such as estimation is accurate asymptotically, it performs quite poorly for our real-world networks.

To approximate $\lambda_1$, we use the formula provided by Chung et al. [2003] for the case of unipartite graphs with a specified degree sequence: $\lambda_1 \approx \mathbb{E}[d_i^2]/\mathbb{E}[d_i]$ where $\mathbb{E}$ is the expectation, and $d_i$ is the degree of node $i$. When working with bipartite networks, we need to account for the degrees of the nodes in the two classes. We use $r_i$ for the degree of the nodes in the first class, and $c_j$ for those in the second. By the same argument of Chung et al. [2003], the approximation for the first eigenvalue of the bipartite graph becomes:

$$\lambda_1 \approx \sqrt{\frac{\mathbb{E}[r_i^2]}{\mathbb{E}[r_i]} \frac{\mathbb{E}[c_j^2]}{\mathbb{E}[c_j]}} ,$$

which already provides an estimate for the configuration model ($\lambda_1^{cm}$), given that the statistics on the degrees are immediate to compute. We want to compute a similar approximation for the Erdős-Rényi case. One

could simply assume that both $r_i$ and $c_i$ are binomially distributed, with parameters $m$ and $p = L/(nm)$ for the case of $r_i$ and $n$ and $p$ for the case of $c_i$. This would yield:

$$\lambda_1 \approx \sqrt{\frac{m^2p^2 + mp(1-p)}{mp} \frac{n^2p^2 + np(1-p)}{np}} = \sqrt{(mp + (1-p))(np + (1-p))} \, .$$

While this approximation works well for sufficiently high connectances $p$, it becomes inaccurate when the network has few connections. Because we are only considering connected networks, each row (column) must contain at least one connection (*i.e.*, $r_i \geq 1$, $c_j \geq 1$ for all $i$ and $j$. To improve the approximation, we account for this fact directly. In particular, the degree of each row follows the random variable $1 + x_r$ where $x_r$ is binomially distributed with parameters $m-1$ and $\tilde{p}_r$, where the "excess connectance" $\tilde{p}_r = (L-n)/(n-m)$. We therefore obtain $\mathbb{E}[r_i] = 1 + \mathbb{E}[x_r] = 1 + (m-1)\tilde{p}_r$ and $\mathbb{E}[r_i^2] = \mathbb{E}[(1+x_r)^2] = 1 + 2\mathbb{E}[x_r] + \mathbb{E}[x_r^2]$. Substituting, we get $\mathbb{E}[r_i^2] = 1 + 2(m-1)\tilde{p}_r + (m-1)^2\tilde{p}_r^2 + (m-1)\tilde{p}_r(1-\tilde{p}_r)$. Performing the same calculation with the columns, we finally obtain:

$$\lambda_1^{er} \approx \sqrt{\frac{1 + (m-1)\tilde{p}_r(3 - \tilde{p}_r + (m-1)\tilde{p}_r)}{1 + (m-1)\tilde{p}_r} \times \frac{1 + (n-1)\tilde{p}_c(3 - \tilde{p}_c + (n-1)\tilde{p}_c)}{1 + (n-1)\tilde{p}_c}} \, ,$$

which performs better when connections are few, and converges to the previous estimate when $p$ is sufficiently large.

For completeness, we also compute the approximation for the case of a random semi-regular bipartite graph, in which all nodes in the same class have the same, average degree, and therefore $\mathbb{E}[r_i^2] = \mathbb{E}[r_i]^2$:

$$\lambda_1^{reg} \approx \sqrt{mpnp} = p\sqrt{nm} \, .$$

Having established the approximations for $\lambda_1$, we turn to $\lambda_2$.

The distribution of the singular values of an Erdős-Rényi random bipartite graph are expected to be formed by a bulk of singular values, following the Marchenko-Pastur distribution [Marchenko and Pastur, 1967], and a single outlier, accounting for the fact that the coefficients of matrix $\mathbf{B}$ have nonzero mean.

If $\mathbf{X}$ is an $n \times m$ matrix whose coefficients are i.i.d. random variables with mean zero and variance $s^2/n$, the largest singular value of $\mathbf{X}$ is approximately $\sigma_1 \approx s^2(1 + \sqrt{m/n})^2$. In our case, we want to compute the variance $s^2$ once we removed the mean ($p$). The second singular value of $\mathbf{B}$ should therefore be $\sigma_2 \approx np(1-p)(1 + \sqrt{m/n})^2$. We therefore obtain the estimate:

$$\lambda_2^{mp} \approx \left(1 + \sqrt{\frac{m}{n}}\right) \sqrt{np(1-p)}$$

## S4.3 Other network properties

We have also recorded a number of popular networks metrics, that can be used to explore the data set. Notice however that for many of these metrics, the correlation with size and fill of the matrix is unkown.

### S4.3.1 Large scale properties

- modularity[Newman, 2006] of the network (where computationally feasible)
- nestedness of the network (where computationally feasible), as measured by:
  - nestedness temperature [Atmar and Patterson, 1993]
  - NODF [Almeida-Neto et al., 2008]
  - the overlap measure of nestedness [Strona and Veech, 2015]
  - The spectral radius [Staniczenko et al., 2013]

#### S4.3.2 Motif-related properies

The counts of some small subgraphs present in the network can be computed directly from degree sequence:

- H2: $\sum_i \binom{d_i}{2}$

  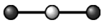

- H3: $(\boldsymbol{d}_{row} - 1) \times \mathbf{B} \times (\boldsymbol{d}_{col} - 1)$

  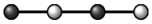

- H4: $\sum_i \binom{d_i}{3}$

  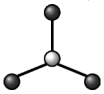

- H17: $\sum_i \binom{d_i}{4}$

  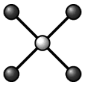

#### S4.3.3 Measures of centrality

We collected several measures of network centralization based on the following centrality measures:

- betweenness centrality
- closeness centrality
- eigenvector centrality

These were calculated using the 'igraph' package [Csardi and Nepusz, 2006] in 'R' [R Core Team, 2018]. Centralization is defined to be the sum of the normalized centrality scores of each node in the network. More precisely, the centralization $C$ of graph $G$ is

$$C(G) = \sum_v \left( \max_w (c(w)) - c(v) \right),$$

where $c(v)$ is the centrality of node $v$.

#### S4.3.4 Clustering coefficient

Clustering in bipartite networks has traditionally been approached in one of two ways. First, one can use traditional measures of clustering (the proportion of completed triangles, or the proportion of a node's connections that are also connected to one another) on one of the two unipartite projections of the bipartite network [Zhang et al., 2008, Opsahl, 2013]. Second, one can extend the foundational logic of clustering to look at paths of length four, rather than the original length three [Lind et al., 2005, Zhang et al., 2008, Opsahl, 2013].

Opsahl [2013] provides the most defensible interpretation of clustering for bipartite graphs, and details the expected value under Erdős-Rényi randomization, which we utilize in our analysis. Importantly, however, there are still significant limitations in this approach. First, this formulation depends on the orientation of the incidence matrix (*i.e.* which node class are rows and which are columns), identifying node classes as either primary or secondary, which is a subjective decision for many of the datasets being analyzed here. Second, as this technique involves counting (or at least sampling) chains of length four, it rapidly becomes computationally infeasible for moderately-sized networks. And third, the normalization proposed by Opsahl [2013] does not completely remove the relationship between size and fill for our data.

#### S4.3.5 Other properties

- network diameter (maximum distance between two nodes)
- average path length between nodes of the network
- degree heterogeneity in each of the row/column nodes (the mean degree squared divided by the mean of the squared degrees)
- degree assortativity (the correlation of degre between connected nodes)

# S5 A Note on Methodology

For the main text, we restricted our analysis in several ways. Some of these choices were dictated by clarity of presentation, while others were more principled. For instance, in the main-text figures, we used a subset of the collected metrics for clarity of presentation. Though we believe a simple solution (*i.e.*, one that utilizes relatively few measures of network structure) would be a more aesthetic solution, this is not a necessary property of a method that can classify ecological interaction networks. On the other hand, our choice to fit the PCA to the non-ecological data stemmed from more principled concerns.

First, not fitting the ecological data directly reduces the risk of overfitting our model to the particular ecological data being used.

Second, by utilizing a wide range of network types, we are able to create a more complete map of the possible space of bipartite network structures. Thus, when the bipartite networks are overlaid onto this space, we have a clearer idea of the general structure of this class as a whole. In every case we considered, the ecological networks most closely aligned with crime networks, suggesting some similarities in structure that might be worth further investigation.

Finally, a more logistical concern stemmed from the quality of currently available ecological data. Due in part to the difficulty of collecting ecological data, and in part to the fact that there are few cases of a single scientist publishing multiple networks, we are faced with a greater amount of noise in the ecological networks, compared with the other data sets. This noise can come from the study system itself (even collecting small ecological network data can be laborious), but also from more systemic sources. For instance, different networks depicting the same type of interaction will nevertheless have different sampling methodologies and span a large geographic and temporal range. Thus, utilizing the more highly and consistently replicated non-ecological data seemed to be a way of avoiding this variation/noise from disrupting the broader approach. In a later section of this supplement, we perform a PCA restricted to these data for comparison.

Each biplot throughout this text was constructed in a similar way: first the data were divided into a training set of 100 (or the size of the smallest network class (whichever was larger) networks from each class, and a testing/fitting set consisting of the remaining networks in teh dataset. A principal component (PC) space was constructed using the training set (fig. A, left panel) and this space was kept constant for each remaining figure, including the right panel of fig. A where the testing data for the PC space used in the main text are plotted. In later sections, we demonstrate alternative PC spaces stemming from including different/additional metrics and/or data.

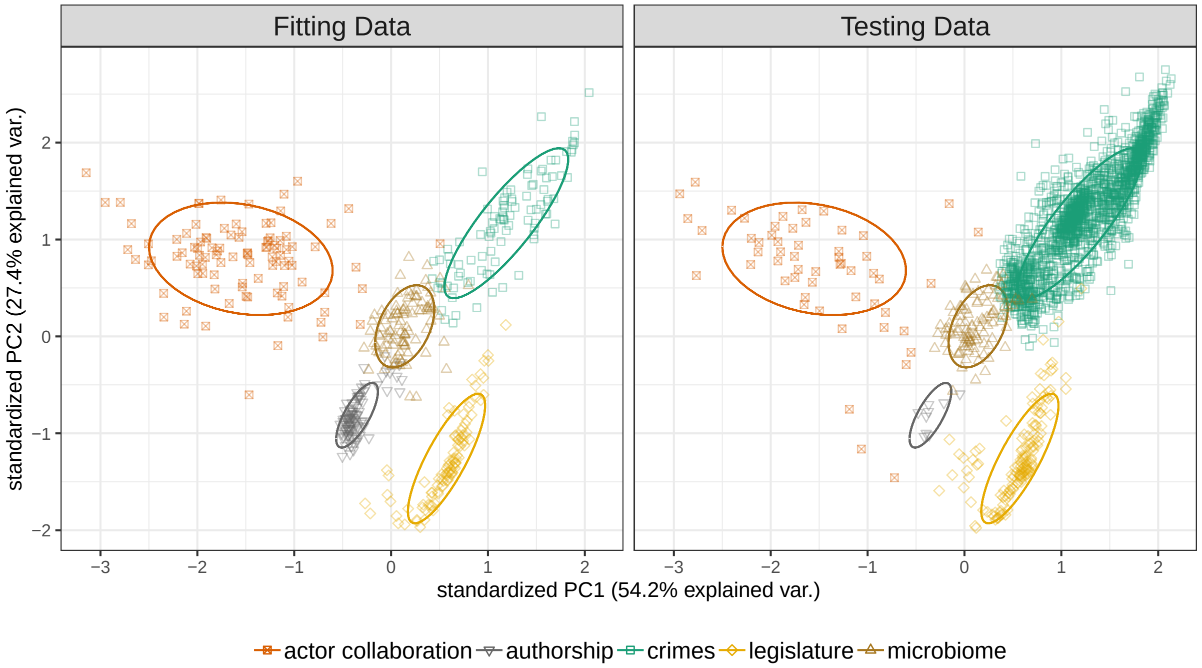


Figure A: A Principal Component Analysis (PCA) biplot of the first two principal components. Left: each point indicates a single network and the ellipses are drawn to contain ≈ 68% of the points in each network type, *i.e.*, one standard deviation if the points were to follow a bivariate normal distribution. Right: all remaining webs in each category projected onto the principal component space defined by the left pane. The fact that the remaining points generally fall within the same region as those used to fit the model is an indication that the model is sufficiently general.

## S6 Uninformative Variation

As argued in the main text, there exists some variation between different networks that is, in general, uninformative with regards to distinguishing between network types. This is because such properties are highly correlated with independent factors such as sampling effort or methodology. In the figures in the main text, we chose metrics in an effort to minimize the influence of these aspects of network structure. In fig. B we provide a visual indication of our degree of success in reducing these effects.

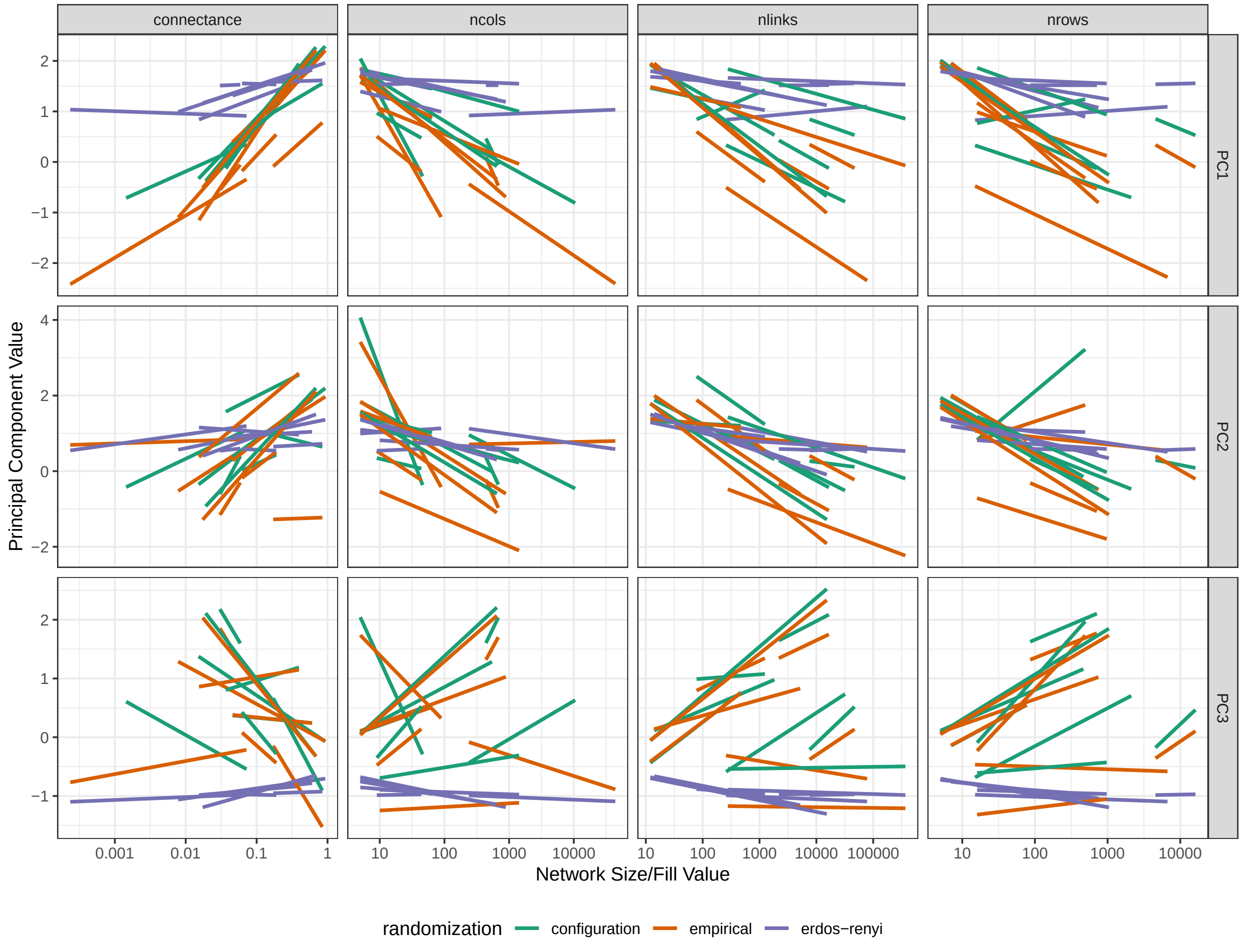


Figure B: Best fitting linear models for each network type, grouped according to randomization. In each plot, the lines indicate the best fitting linear model of the form *PC metric*, where the particular principal component (PC) is indicated by the horizontal facetting and the network metric is indicated by the vertical facetting. In each panel, each network type is represented by three lines, corresponding to the empirical networks (orange) and the sets of Erdős-Rényi and configuration model randomizations (purple and green, respectively). The reduced slopes for the Erdős-Rényi regressions indicate greater success at reducing the effect of size and fill on our map of network types.

## S7 Alternative Metrics

In this section, we provide analogous figures to those in the main text which include all colleced metrics for comparison. Note that, because several of these metrics have strong correlations with size and fill, we do not see the desired collapsing behavior in the randomized networks (figs. C to F).

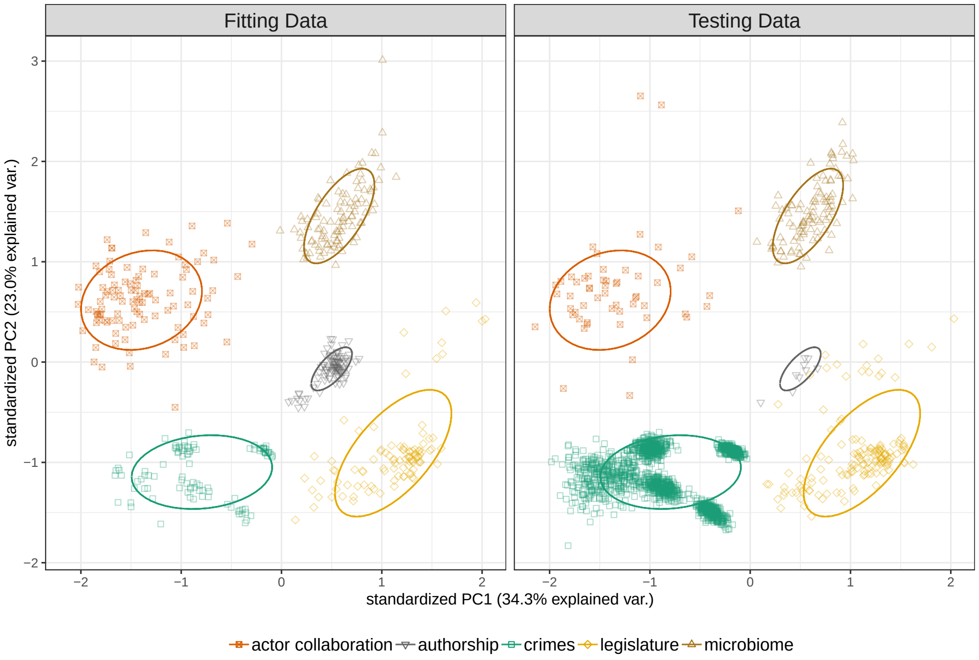


Figure C: As fig. A, but using all metrics.

Note that, while we obtain even better separation for the non-ecological networks, the mutualistic and antagonistic networks still fall nearly on top of one another. These figures also reveal a clear tradeoff between specificity and generality: this better separation for non-ecological data comes at the cost of being able to tell randomized networks apart, meaning that at least some of our ability to distinguish network types is an artifact of the different sizes and connectances of the different classes. To avoid this, one option would be to create empirical z-scores for each of the metrics employed, by taking several randomizations and computing mean and variances. The downside of this approach is that randomizing the large networks is computationally very challenging.

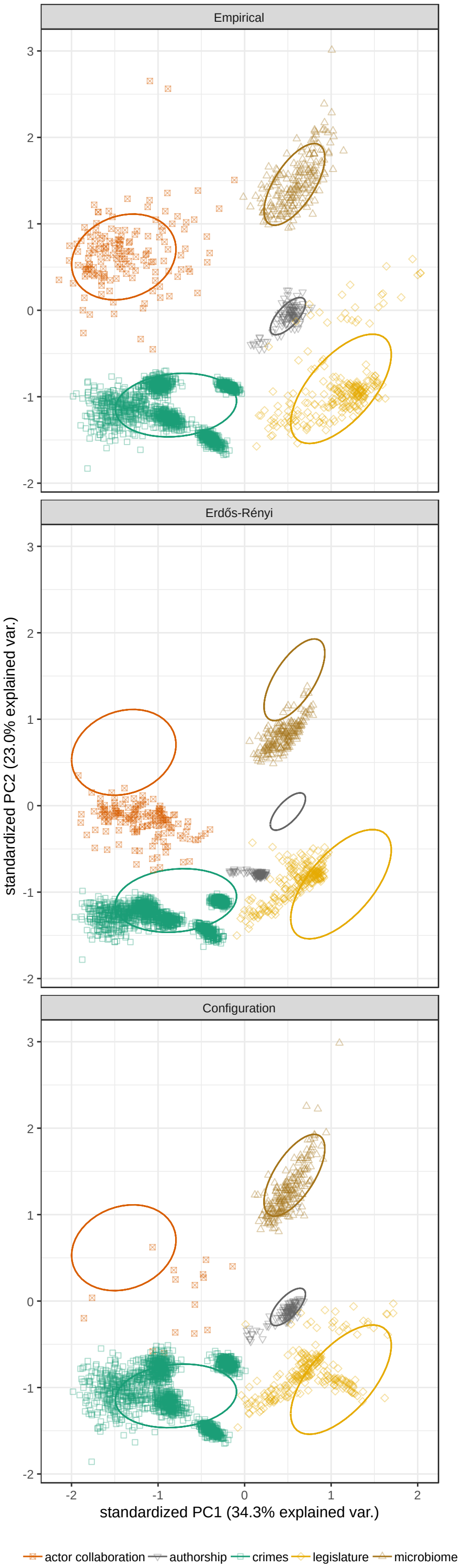


Figure D: As Figure 1 of the main text, but using all metrics.

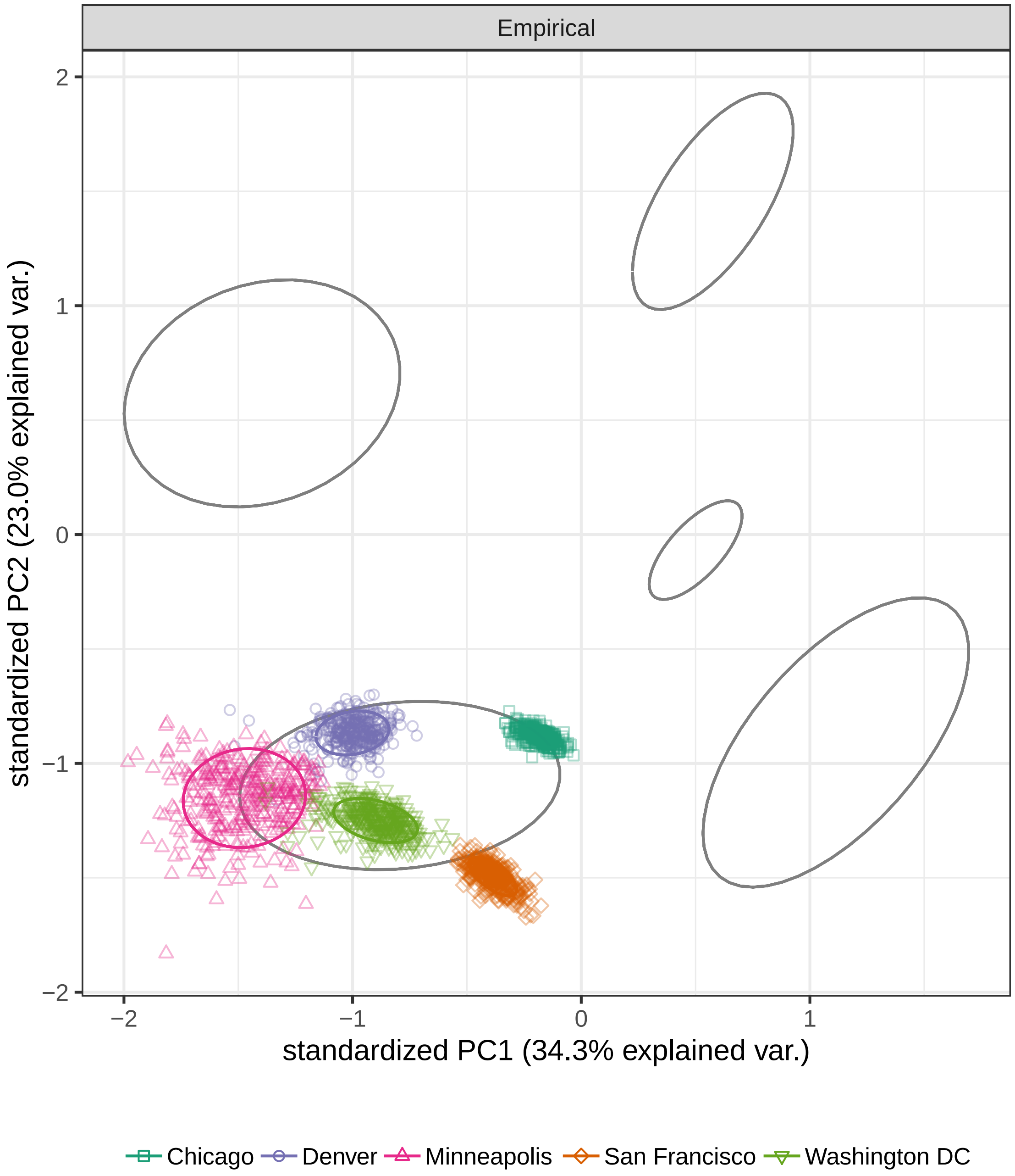


Figure E: As Figure 2 of the main text, but using all metrics.

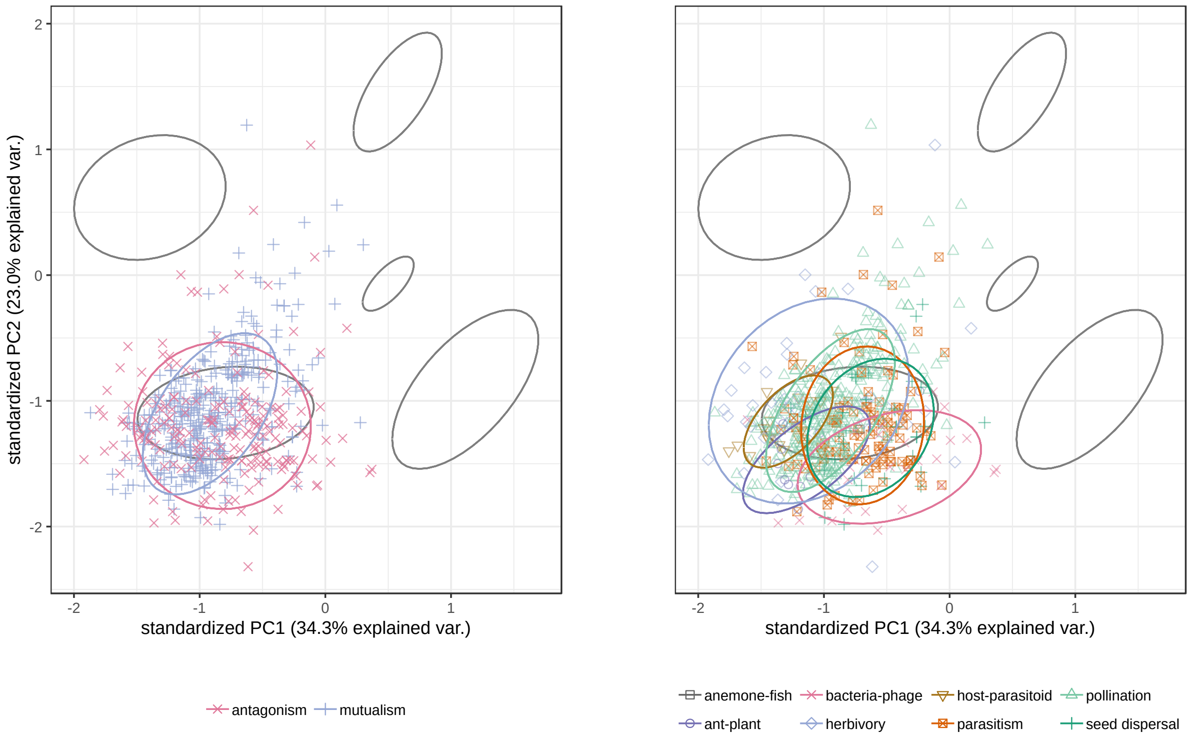


Figure F: As Figure 3 of the main text, but using all metrics.

# S8 Focusing on Nestedness and Modularity

In table A, we report the statistics run on the results presented in Figure 5 of the main text. To compare the distributions, we followed [Thébault and Fontaine, 2010] in using Welch's t-test (which accounts for different sample sizes and variances; [Welch, 1947]).

As an alternative to Figure 5 in the main text, we could investigate the role of Nestedness and Modularity in distinguishing ecological networks using PCA (fig. G). Here, we ran a PCA just on the ecological data using just measures of nestedness and modularity as input values. As before, there is no separation between interaction type.

Table A: Nestedness and modularity statistical comparison

| Null Model | Metric | mean (size) antagonism | mean (size) mutualism | degrees of freedom | P-Value |
|---|---|---|---|---|---|
| Configuration Model | NODF[1] | 0.0978(173) | 0.0839(245) | 403.0649 | 0.370 |
| | Overlap[2] | −0.2188(173) | −0.2536(245) | 214.4697 | 0.010 |
| | $\rho$[3] | −0.0178(173) | 0.0135(245) | 414.6372 | 0.827 |
| | Temperature[4] | 0.012(173) | 0.0618(245) | 271.7934 | 0.789 |
| | Modularity[5] | 0.1814(173) | 0.1949(245) | 402.6117 | 0.093 |
| Erdős-Rényi | NODF[1] | −0.2391(188) | 0.6974(248) | 433.7538 | $< 0.001$ |
| | Overlap[2] | 39.4128(188) | −1.4504(248) | 192.7469 | 0.377 |
| | $\rho$[3] | −0.0623(188) | 0.6483(248) | 423.9124 | 0.152 |
| | Temperature[4] | 0.0248(188) | −0.5723(248) | 371.7239 | 0.176 |
| | Modularity[5] | 0.0158(188) | −0.0498(248) | 282.2995 | 0.101 |

[1] Almeida-Neto et al. [2008], [2] Strona and Veech [2015], [3] Staniczenko et al. [2013], [4] Atmar and Patterson [1993], [5] Newman [2006]

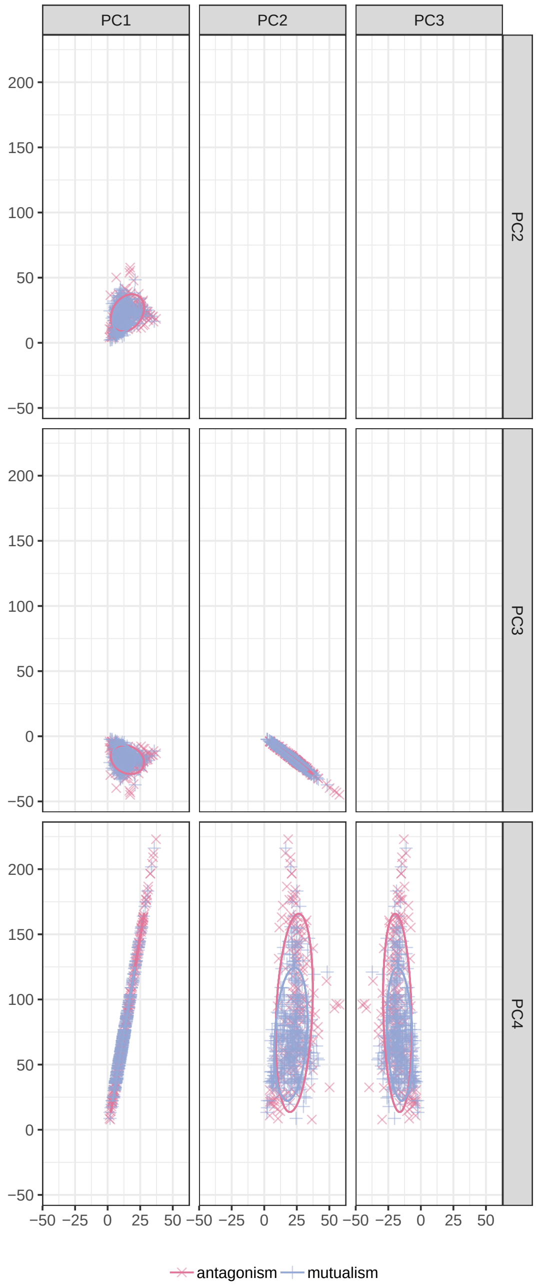


Figure G: PCA using only measures of nestedness and modularity.

## S9 Alternative Ecological Data

In an attempt to address the large variation in ecological networks, in fig. H we replot the ecological networks using only cases where there are multiple networks published by the same group. Often, these are networks in similar environmental conditions and were collected using similar methods over a relatively constrained amount of time. Moreover, because networks that meet these criteria also tend to be more recently published, they also tend to be larger, on average, than older, lower-quality networks.

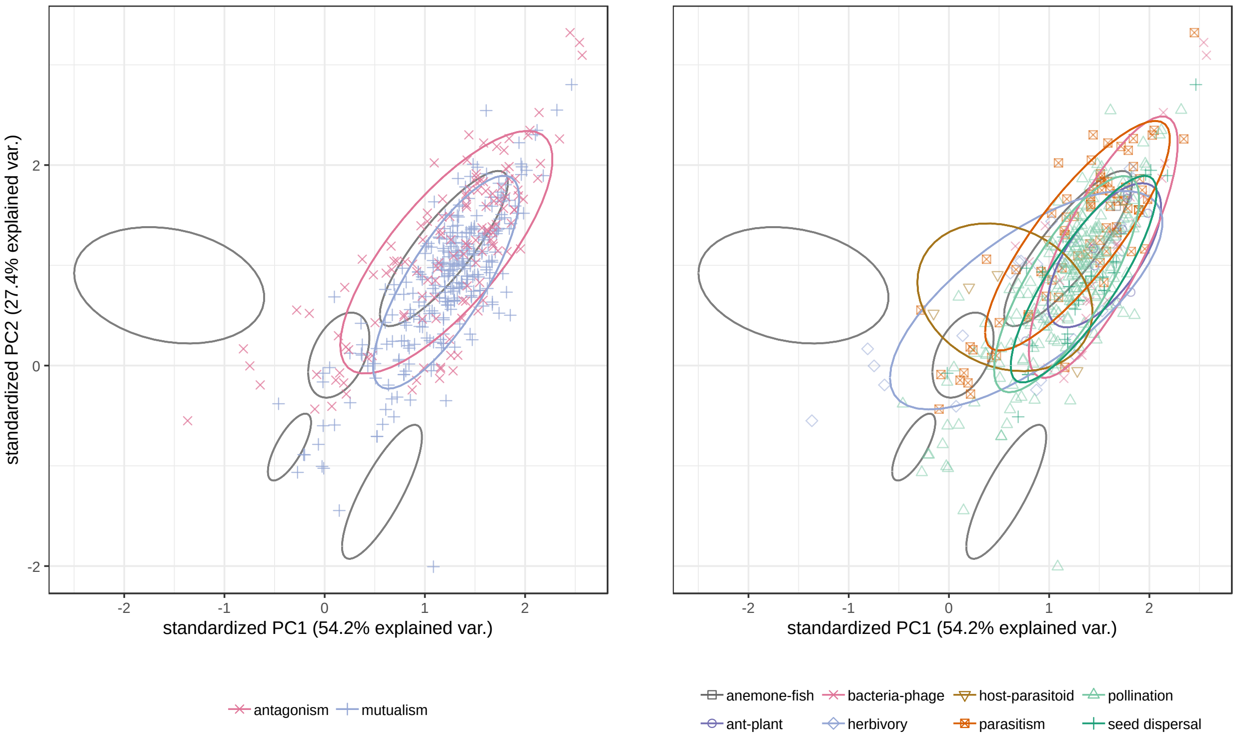


Figure H: As Figure 3 of the main text, but using only multiple networks collected by the same groups.

## S9.1 More Dimensions

Perhaps there is separation in our results, but not within the first two principal component dimensions. In fig. I we plot each pairwise combination of dimensions for the PCA from the main text (Note that here we are only plotting the ecological networks, but we are still projecting them into the space defined by the PCA defined by the non-ecological networks):

and the same for each pairwise combination of the first five dimensions of the full PCA presented in this supplement (fig. J):

For all cases, the level of overlap is substantially greater than that seen between the non-ecological networks.

Again, it is difficult to distinguish the clusters.

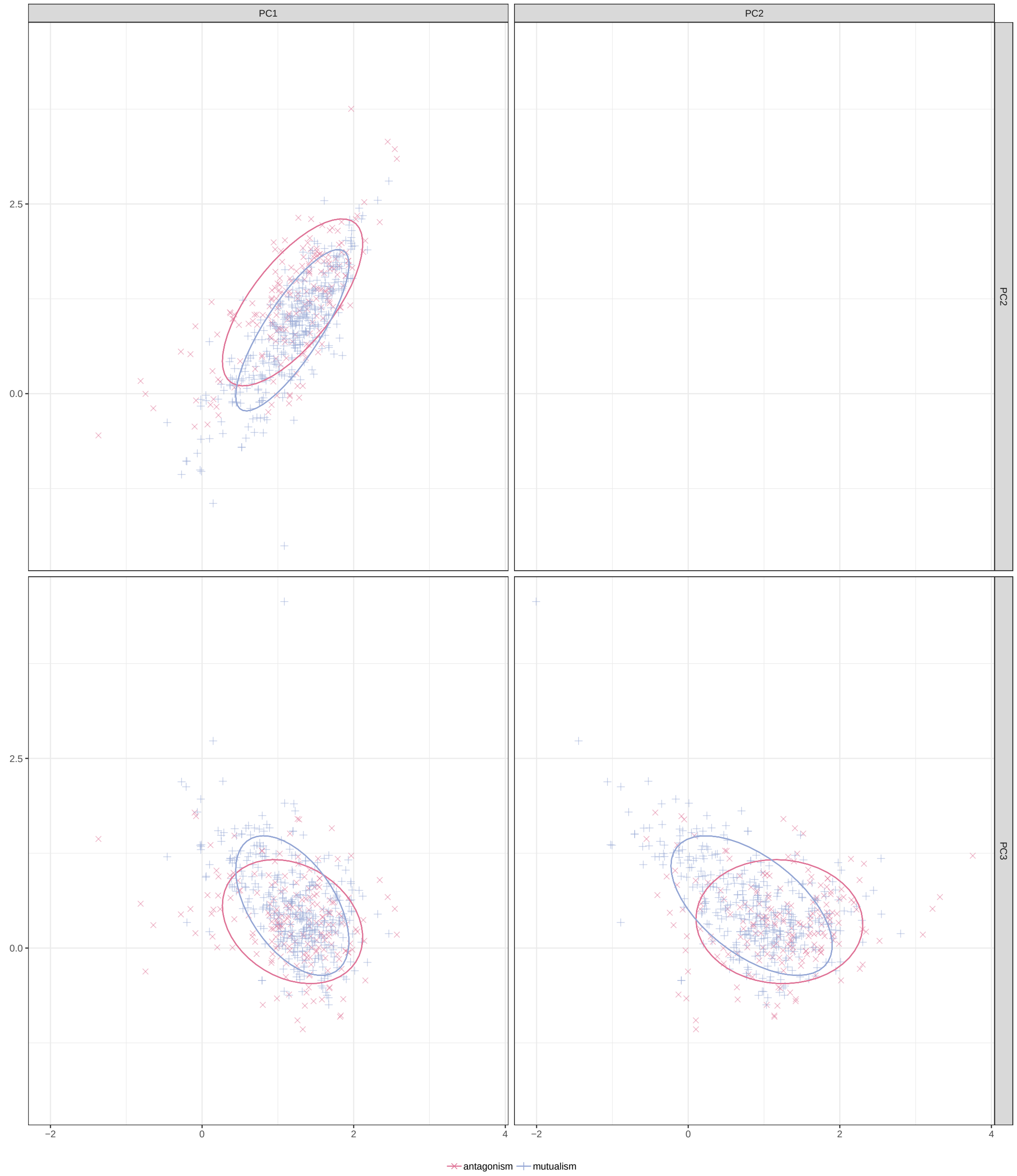


Figure I: Biplots for the three combinations of axes.

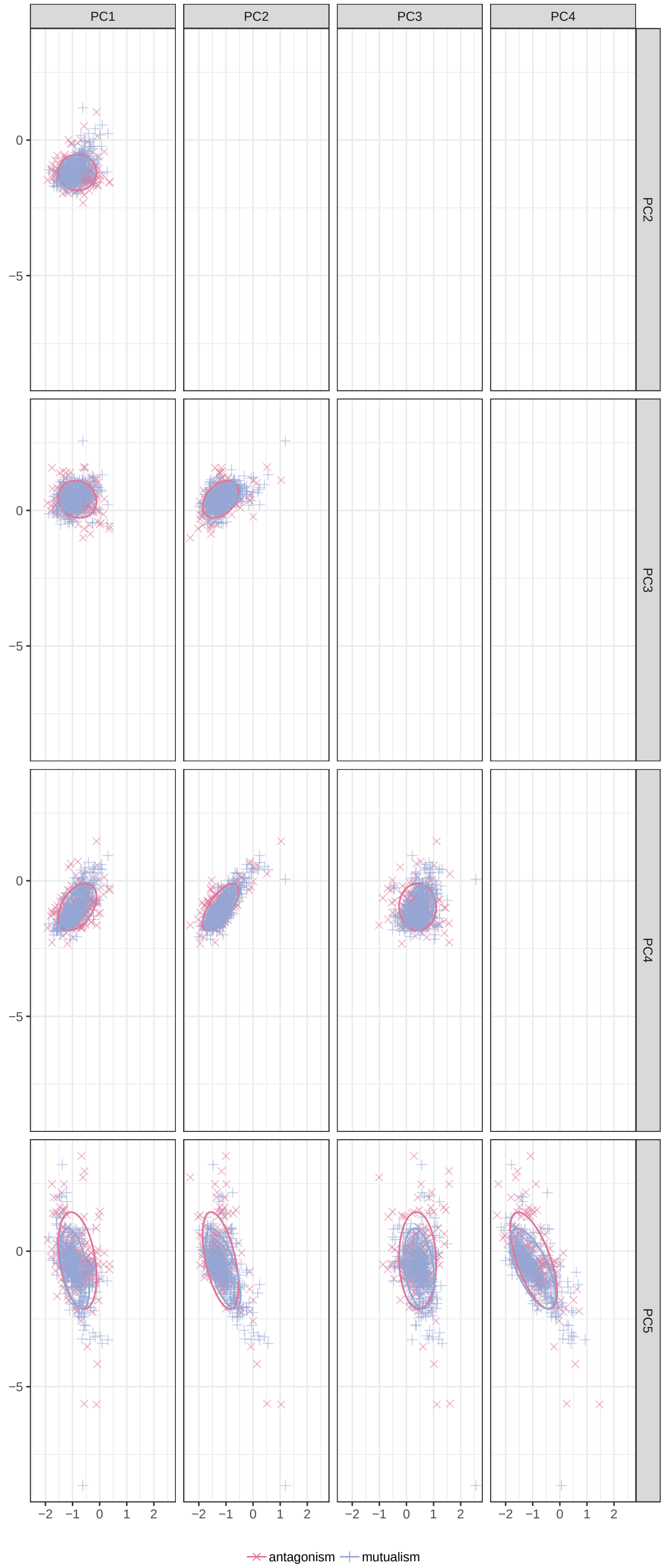


Figure J: Biplots for the first five components, using all metrics.

## S10 Including Ecological Data in the Fitting

Perhaps the lack of separation is due to our process of not including the ecological data in the model construction, thus leading to principal components which were not constructed to explain the variation within ecological networks. Though we believe this choice is stil justified (see the Note on Methodology (section S5), we include figures resulting from this approach here for comparison (again mirroring figures from the main text; figs. K and L):

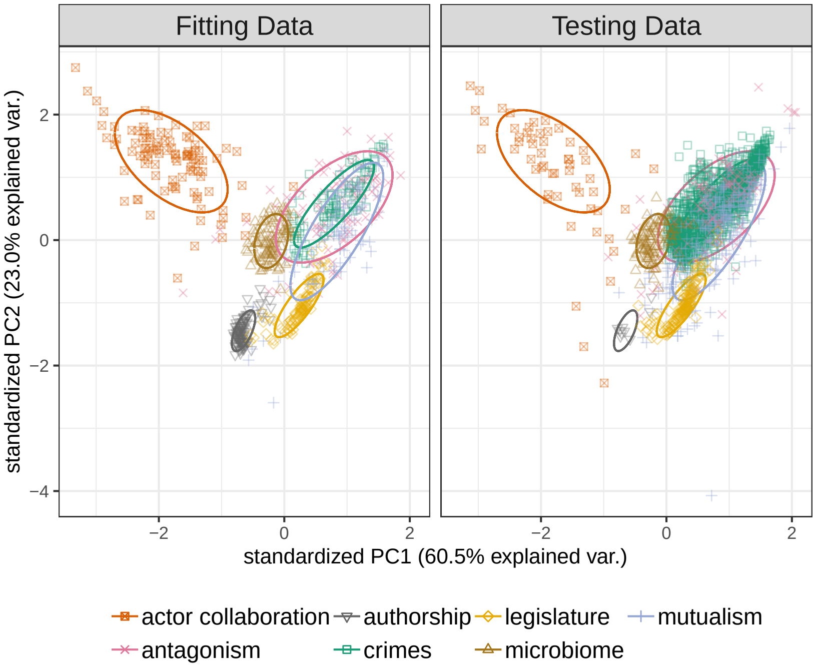


Figure K: As fig. A, but using using a principal component space defined with fitting data that includes ecological interaction networks.

As before, there is no separation between mutualistic and antagonisitic networks, which fall over the microbiome and crime networks.

Similar results are obtained when restricting to the "high quality" ecological datasets (note here that we had to reduce our sample size for the fitting dataset to the size of the smallest category: antagoinstic with 48 networks; figs. M and N)):

and when using the full set of metrics collected (figs. O and P):

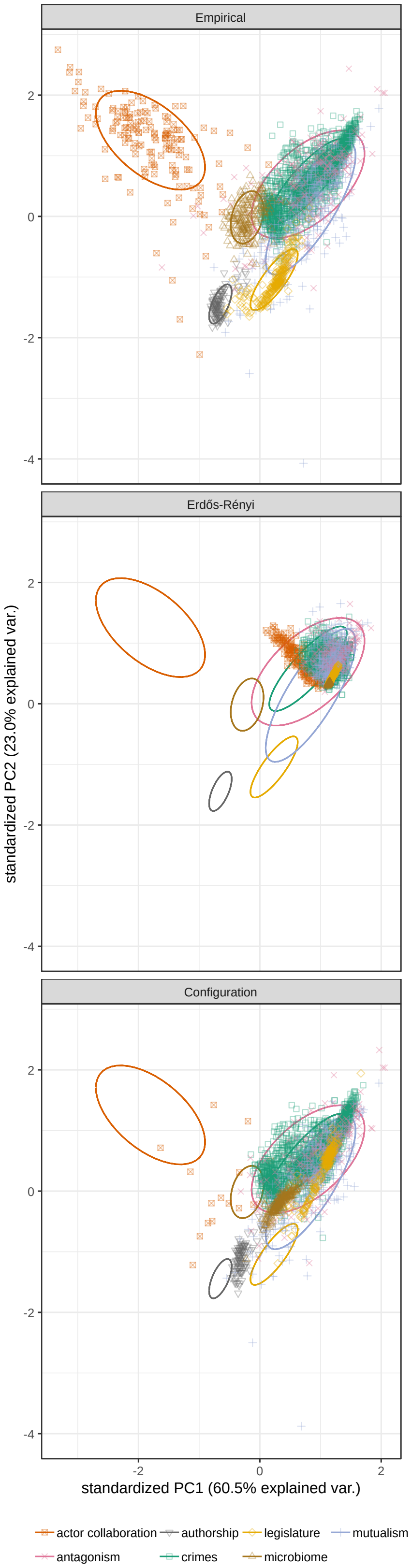


Figure L: As Figure 1 of the main text, but using using a principal component space defined with fitting data that includes ecological interaction networks.

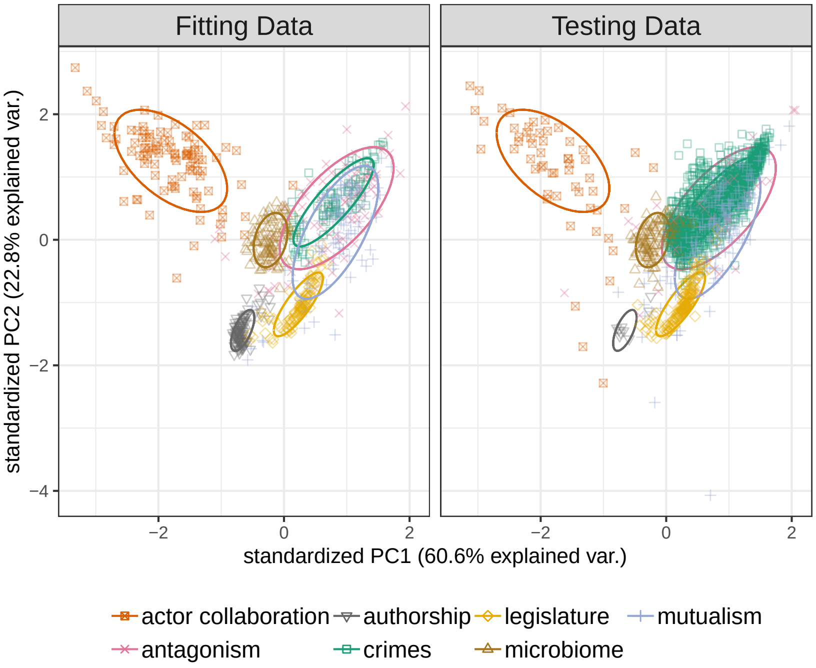


Figure M: As fig. A, but using using a principal component space defined with fitting data that includes high-quality ecological interaction networks.

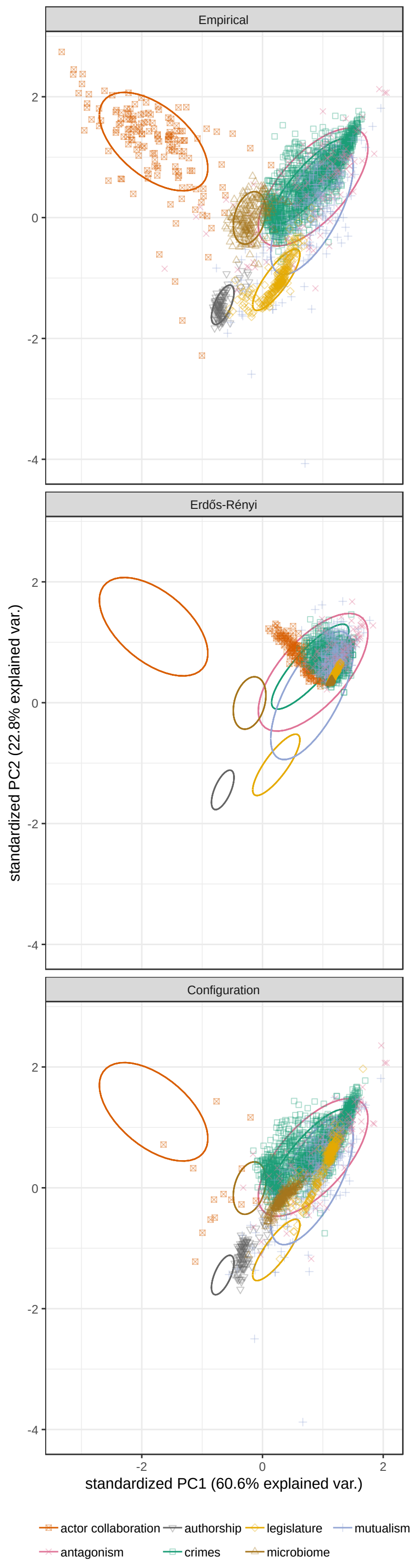


Figure N: As Figure 1 of the main text, but using using a principal component space defined with fitting data that includes high-quality ecological interaction networks.

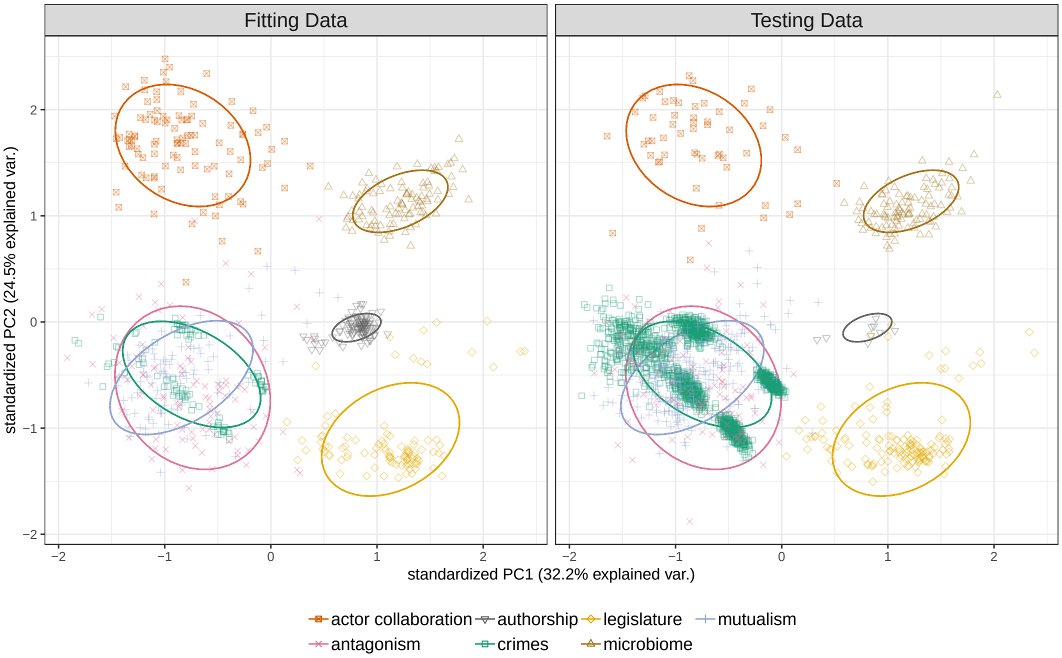


Figure O: As fig. A, but using using a principal component space defined with fitting data that includes high-quality ecological interaction networks and utilizing the full suite of metrics.

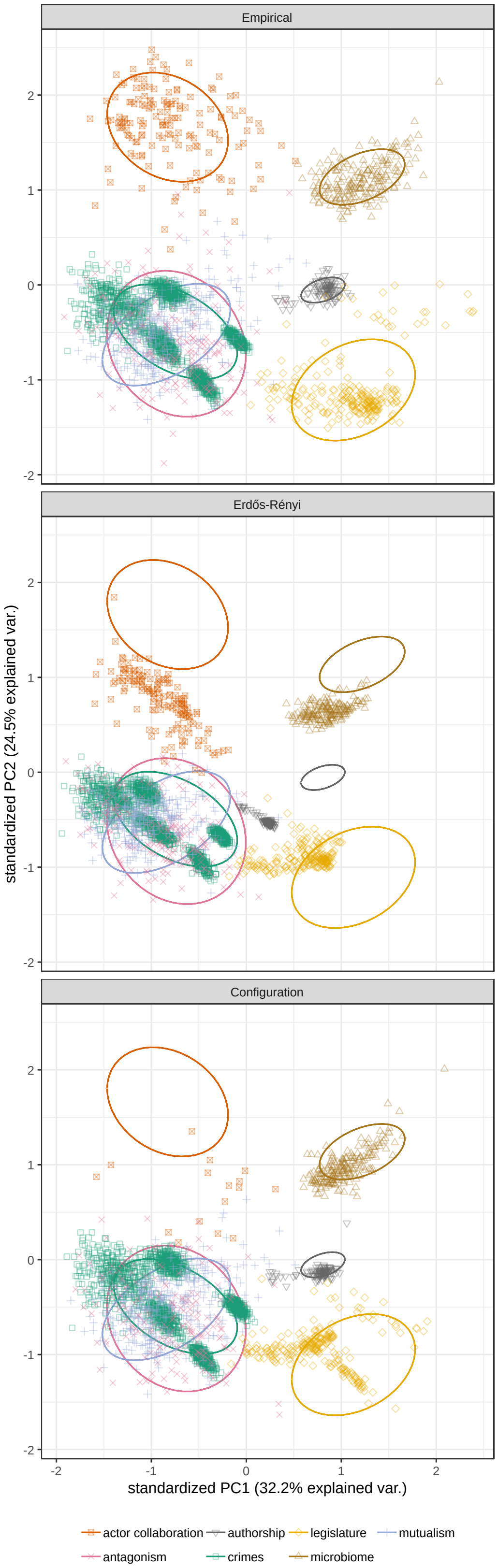


Figure P: As Figure 1 of the main text, but using using a principal component space defined with fitting data that includes high-quality ecological interaction networks and utilizing the full suite of metrics.

# S11 Machine Learning Approaches

As noted in the main text, Principal Component Analysis is not necessarily the best tool for addressing this problem, but it does have some advantages. In this section, we apply two alternative approaches from the machine learning literature: Random Forests and Gradient Boosted Regression Trees. In both cases, the degree of success is often represented through confusion matrices, where the predicted classification of the testing data is compared to the known correct classification. This provides a clear measure of sensitivity and specificity, but does not produce the visual map we present as a measure of PCA's success. Moreover, with these approaches, it doesn't make sense to do the "ecological-data-agnostic" approach we apply in the main text — all classes must be present throughout the analysis.

In all cases below, we are able to get high specificity for both antagonistic and mutualistic ecological interaction networks, but we fall short on sensitivity. That is, when the models categorize something as an antagonistic interaction network, we can be quite sure that that classification is correct, yet the model is less successful at ensuring that all antagonistic ecological networks are detected.

## S11.1 Using same metrics as main text

### S11.1.1 Random Forest

Table B: Random forest results using metrics from the main text.

| | actor collab. | antagonism | authorship | crimes | legislature | microbiome | mutualism |
|---|---|---|---|---|---|---|---|
| actor collab. | 34 | 2 | 0 | 0 | 0 | 0 | 0 |
| antagonism | 2 | 7 | 0 | 2 | 1 | 0 | 3 |
| authorship | 1 | 0 | 26 | 0 | 0 | 0 | 1 |
| crimes | 0 | 27 | 0 | 427 | 0 | 0 | 38 |
| legislature | 0 | 0 | 0 | 0 | 60 | 1 | 3 |
| microbiome | 1 | 1 | 0 | 0 | 0 | 50 | 0 |
| mutualism | 1 | 11 | 1 | 25 | 0 | 0 | 43 |
| Sensitivity | 0.87 | 0.15 | 0.96 | 0.94 | 0.98 | 0.98 | 0.49 |
| Specificity | 1.00 | 0.99 | 1.00 | 0.79 | 0.99 | 1.00 | 0.94 |
| Pos Pred Value | 0.94 | 0.47 | 0.93 | 0.87 | 0.94 | 0.96 | 0.53 |
| Neg Pred Value | 0.99 | 0.95 | 1.00 | 0.90 | 1.00 | 1.00 | 0.93 |
| Precision | 0.94 | 0.47 | 0.93 | 0.87 | 0.94 | 0.96 | 0.53 |
| Recall | 0.87 | 0.15 | 0.96 | 0.94 | 0.98 | 0.98 | 0.49 |
| F1 | 0.91 | 0.22 | 0.95 | 0.90 | 0.96 | 0.97 | 0.51 |
| Prevalence | 0.05 | 0.06 | 0.04 | 0.59 | 0.08 | 0.07 | 0.11 |
| Detection Rate | 0.04 | 0.01 | 0.03 | 0.56 | 0.08 | 0.07 | 0.06 |
| Detection Prevalence | 0.05 | 0.02 | 0.04 | 0.64 | 0.08 | 0.07 | 0.11 |
| Balanced Accuracy | 0.93 | 0.57 | 0.98 | 0.87 | 0.99 | 0.99 | 0.72 |

### S11.1.2 Gradient Boosted Regression Trees

Table C: Gradient boosted regression tree results using metrics from the main text.

| | actor collab. | antagonism | authorship | crimes | legislature | microbiome | mutualism |
|---|---|---|---|---|---|---|---|
| actor collab. | 35 | 2 | 0 | 0 | 0 | 0 | 0 |
| antagonism | 0 | 1 | 0 | 1 | 0 | 0 | 3 |
| authorship | 2 | 1 | 27 | 0 | 0 | 0 | 2 |
| crimes | 0 | 31 | 0 | 436 | 1 | 0 | 47 |
| legislature | 0 | 0 | 0 | 0 | 60 | 1 | 3 |
| microbiome | 1 | 2 | 0 | 0 | 0 | 50 | 0 |
| mutualism | 1 | 11 | 0 | 17 | 0 | 0 | 33 |
| Sensitivity | 0.90 | 0.02 | 1.00 | 0.96 | 0.98 | 0.98 | 0.38 |
| Specificity | 1.00 | 0.99 | 0.99 | 0.75 | 0.99 | 1.00 | 0.96 |
| Pos Pred Value | 0.95 | 0.20 | 0.84 | 0.85 | 0.94 | 0.94 | 0.53 |
| Neg Pred Value | 0.99 | 0.94 | 1.00 | 0.93 | 1.00 | 1.00 | 0.92 |
| Precision | 0.95 | 0.20 | 0.84 | 0.85 | 0.94 | 0.94 | 0.53 |
| Recall | 0.90 | 0.02 | 1.00 | 0.96 | 0.98 | 0.98 | 0.38 |
| F1 | 0.92 | 0.04 | 0.92 | 0.90 | 0.96 | 0.96 | 0.44 |
| Prevalence | 0.05 | 0.06 | 0.04 | 0.59 | 0.08 | 0.07 | 0.11 |
| Detection Rate | 0.05 | 0.00 | 0.04 | 0.57 | 0.08 | 0.07 | 0.04 |
| Detection Prevalence | 0.05 | 0.01 | 0.04 | 0.67 | 0.08 | 0.07 | 0.08 |
| Balanced Accuracy | 0.95 | 0.51 | 1.00 | 0.85 | 0.99 | 0.99 | 0.67 |

## S11.2 Using the full set of metrics

As with the PCA results listed above, using the full set of metrics improves our ability to distinguish ecological networks, but still not to the level we obtain with our nonecological datasets.

### S11.2.1 Random Forest

Table D: Random forest results using all metrics.

| | actorcollaboration | antagonism | authorship | crimes | legislature | microbiome | mutualism |
|---|---|---|---|---|---|---|---|
| actorcollaboration | 38 | 1 | 0 | 0 | 0 | 0 | 0 |
| antagonism | 1 | 21 | 0 | 1 | 1 | 0 | 11 |
| authorship | 0 | 0 | 27 | 0 | 0 | 0 | 0 |
| crimes | 0 | 10 | 0 | 446 | 1 | 0 | 7 |
| legislature | 0 | 0 | 0 | 0 | 59 | 0 | 1 |
| microbiome | 0 | 0 | 0 | 0 | 0 | 51 | 0 |
| mutualism | 0 | 17 | 0 | 7 | 0 | 0 | 72 |
| Sensitivity | 0.97 | 0.43 | 1.00 | 0.98 | 0.97 | 1.00 | 0.79 |
| Specificity | 1.00 | 0.98 | 1.00 | 0.94 | 1.00 | 1.00 | 0.96 |
| Pos Pred Value | 0.97 | 0.60 | 1.00 | 0.96 | 0.98 | 1.00 | 0.75 |
| Neg Pred Value | 1.00 | 0.96 | 1.00 | 0.97 | 1.00 | 1.00 | 0.97 |
| Precision | 0.97 | 0.60 | 1.00 | 0.96 | 0.98 | 1.00 | 0.75 |
| Recall | 0.97 | 0.43 | 1.00 | 0.98 | 0.97 | 1.00 | 0.79 |
| F1 | 0.97 | 0.50 | 1.00 | 0.97 | 0.98 | 1.00 | 0.77 |
| Prevalence | 0.05 | 0.06 | 0.03 | 0.59 | 0.08 | 0.07 | 0.12 |
| Detection Rate | 0.05 | 0.03 | 0.03 | 0.58 | 0.08 | 0.07 | 0.09 |
| Detection Prevalence | 0.05 | 0.05 | 0.03 | 0.60 | 0.08 | 0.07 | 0.12 |
| Balanced Accuracy | 0.99 | 0.70 | 1.00 | 0.96 | 0.98 | 1.00 | 0.88 |

#### S11.2.2 Gradient Boosted Regression Trees

Table E: Gradient boosted regression tree results using all metrics.

| | actor collab. | antagonism | crimes | legislature | mutualism |
|---|---|---|---|---|---|
| actor collab. | 3 | 0 | 0 | 0 | 0 |
| antagonism | 0 | 24 | 2 | 0 | 5 |
| crimes | 0 | 13 | 450 | 1 | 12 |
| legislature | 0 | 0 | 0 | 8 | 0 |
| mutualism | 0 | 11 | 1 | 0 | 72 |
| Sensitivity | 1.00 | 0.50 | 0.99 | 0.89 | 0.81 |
| Specificity | 1.00 | 0.99 | 0.83 | 1.00 | 0.98 |
| Pos Pred Value | 1.00 | 0.77 | 0.95 | 1.00 | 0.86 |
| Neg Pred Value | 1.00 | 0.96 | 0.98 | 1.00 | 0.97 |
| Precision | 1.00 | 0.77 | 0.95 | 1.00 | 0.86 |
| Recall | 1.00 | 0.50 | 0.99 | 0.89 | 0.81 |
| F1 | 1.00 | 0.61 | 0.97 | 0.94 | 0.83 |
| Prevalence | 0.00 | 0.08 | 0.75 | 0.01 | 0.15 |
| Detection Rate | 0.00 | 0.04 | 0.75 | 0.01 | 0.12 |
| Detection Prevalence | 0.00 | 0.05 | 0.79 | 0.01 | 0.14 |
| Balanced Accuracy | 1.00 | 0.74 | 0.91 | 0.94 | 0.89 |

### S11.3 Directly Parsing Ecological Networks

In the above examples, we compare non-ecological classification to that for ecological networks (as we do in the main text). In principal, such a comparison is not as relevant when using machine learning approaches such as these. Instead, we can just plug in the two (or more) types directly. The results are not qualitatively different from above, but the difficulties of classification are more apparent.

#### S11.3.1 Random Forest

Table F: Random forest results for directly distinguishing ecological network types

| | antagonism | mutualism |
|---|---|---|
| antagonism | 29 | 13 |
| mutualism | 19 | 76 |

| | |
|---|---|
| Sensitivity | 0.60 |
| Specificity | 0.85 |
| Pos Pred Value | 0.69 |
| Neg Pred Value | 0.80 |
| Precision | 0.69 |
| Recall | 0.60 |
| F1 | 0.64 |
| Prevalence | 0.35 |
| Detection Rate | 0.21 |
| Detection Prevalence | 0.31 |
| Balanced Accuracy | 0.73 |

#### S11.3.2 Gradient Boosted Regression Trees

Table G: Gradient boosted regression tree results for directly distinguishing ecological network types

| | antagonism | mutualism |
|---|---|---|
| antagonism | 31 | 12 |
| mutualism | 17 | 77 |

| | |
|---|---|
| Sensitivity | 0.65 |
| Specificity | 0.87 |
| Pos Pred Value | 0.72 |
| Neg Pred Value | 0.82 |
| Precision | 0.72 |
| Recall | 0.65 |
| F1 | 0.68 |
| Prevalence | 0.35 |
| Detection Rate | 0.23 |
| Detection Prevalence | 0.31 |
| Balanced Accuracy | 0.76 |

## S11.4 Directly Parsing Ecological Networks—Subcategorizations

Here, we repeat the above analysis with the subcategorization of ecological interaction network type. As can be seen below, some sub-types tend to have high specificity, while others have high sensitivity, but in no cases are both high as was the case for the non-ecological data.

### S11.4.1 Random Forest

Table H: Random forest results for directly distinguishing subcategorized ecological network types

| | ant-plant | bacteria-phage | host-parasitoid | host-parasite | plant-herbivore | plant-pollinator | seed-disperser |
|---|---|---|---|---|---|---|---|
| ant-plant | 0 | 0 | 0 | 0 | 0 | 0 | 0 |
| bacteria-phage | 0 | 3 | 0 | 0 | 1 | 0 | 1 |
| host-parasitoid | 0 | 0 | 0 | 0 | 0 | 0 | 0 |
| host-parasite | 0 | 2 | 0 | 11 | 1 | 1 | 0 |
| plant-herbivore | 0 | 0 | 1 | 0 | 1 | 1 | 0 |
| plant-pollinator | 2 | 2 | 5 | 15 | 6 | 76 | 6 |
| seed-disperser | 0 | 0 | 0 | 1 | 1 | 0 | 3 |
| Sensitivity | 0.00 | 0.43 | 0.00 | 0.41 | 0.10 | 0.97 | 0.30 |
| Specificity | 1.00 | 0.98 | 1.00 | 0.96 | 0.98 | 0.42 | 0.98 |
| Pos Pred Value | | 0.60 | | 0.73 | 0.33 | 0.68 | 0.60 |
| Neg Pred Value | 0.99 | 0.97 | 0.96 | 0.87 | 0.93 | 0.93 | 0.95 |
| Precision | | 0.60 | | 0.73 | 0.33 | 0.68 | 0.60 |
| Recall | 0.00 | 0.43 | 0.00 | 0.41 | 0.10 | 0.97 | 0.30 |
| F1 | | 0.50 | | 0.52 | 0.15 | 0.80 | 0.40 |
| Prevalence | 0.01 | 0.05 | 0.04 | 0.19 | 0.07 | 0.56 | 0.07 |
| Detection Rate | 0.00 | 0.02 | 0.00 | 0.08 | 0.01 | 0.54 | 0.02 |
| Detection Prevalence | 0.00 | 0.04 | 0.00 | 0.11 | 0.02 | 0.80 | 0.04 |
| Balanced Accuracy | 0.50 | 0.71 | 0.50 | 0.69 | 0.54 | 0.70 | 0.64 |

### S11.4.2 Gradient Boosted Regression Trees

Table I: Gradient boosted regression tree results for directly distinguishing subcategorized ecological network types

| | ant-plant | bacteria-phage | host-parasitoid | host-parasite | plant-herbivore | plant-pollinator | seed-disperser |
|---|---|---|---|---|---|---|---|
| ant-plant | 0 | 0 | 0 | 0 | 0 | 0 | 0 |
| bacteria-phage | 0 | 3 | 0 | 0 | 1 | 0 | 0 |
| host-parasitoid | 0 | 0 | 1 | 0 | 0 | 0 | 0 |
| host-parasite | 0 | 2 | 0 | 13 | 0 | 1 | 0 |
| plant-herbivore | 0 | 0 | 1 | 0 | 2 | 2 | 0 |
| plant-pollinator | 2 | 2 | 4 | 13 | 5 | 74 | 7 |
| seed-disperser | 0 | 0 | 0 | 1 | 2 | 1 | 3 |
| Sensitivity | 0.00 | 0.43 | 0.17 | 0.48 | 0.20 | 0.95 | 0.30 |
| Specificity | 1.00 | 0.99 | 1.00 | 0.97 | 0.98 | 0.47 | 0.97 |
| Pos Pred Value | | 0.75 | 1.00 | 0.81 | 0.40 | 0.69 | 0.43 |
| Neg Pred Value | 0.99 | 0.97 | 0.96 | 0.89 | 0.94 | 0.88 | 0.95 |
| Precision | | 0.75 | 1.00 | 0.81 | 0.40 | 0.69 | 0.43 |
| Recall | 0.00 | 0.43 | 0.17 | 0.48 | 0.20 | 0.95 | 0.30 |
| F1 | | 0.55 | 0.29 | 0.60 | 0.27 | 0.80 | 0.35 |
| Prevalence | 0.01 | 0.05 | 0.04 | 0.19 | 0.07 | 0.56 | 0.07 |
| Detection Rate | 0.00 | 0.02 | 0.01 | 0.09 | 0.01 | 0.53 | 0.02 |
| Detection Prevalence | 0.00 | 0.03 | 0.01 | 0.11 | 0.04 | 0.76 | 0.05 |
| Balanced Accuracy | 0.50 | 0.71 | 0.58 | 0.73 | 0.59 | 0.71 | 0.63 |

# S12 Code availability

All code used to run these analyses was written in 'R' [R Core Team, 2018] with the use of several packages [Csardi and Nepusz, 2006, Dormann et al., 2009, 2008, Dormann, 2011, Wickham, 2017b, Bache and Wickham, 2014, Liaw and Wiener, 2002, Wickham, 2017c, Robinson, 2017, Wickham, 2017b,a, Dahl, 2016]. This code is made available through a public Git repository associated with this project (`http://git.io/f4y4o`).

# References


Mário Almeida-Neto, Paulo Guimaraes, Paulo R Guimarães, Rafael D Loyola, and Werner Ulrich. A consisten metric for nestedness analysis in ecological systems: reconciling concept and measurement. *Oikos*, 117(8): 1227–1239, 2008.

Hisao P Arai and Dwight R Mudry. Protozoan and metazoan parasites of fishes from the headwaters of the parsnip and mcgregor rivers, british columbia: a study of possible parasite transfaunations. *Canadian Journal of Fisheries and Aquatic Sciences*, 40(10):1676–1684, 1983.

Mary T Kalin Arroyo, Richard Primack, and Juan Armesto. Community studies in pollination ecology in the high temperate andes of central chile. i. pollination mechanisms and altitudinal variation. *American journal of botany*, pages 82–97, 1982.

JR Arthur, L Margolis, and HP Arai. Parasites of fishes of aishihik and stevens lakes, yukon territory, and potential consequences of their interlake transfer through a proposed water diversion for hydroelectrical purposes. *Journal of the Fisheries Board of Canada*, 33(11):2489–2499, 1976.

Wirt Atmar and Bruce D Patterson. The measure of order and disorder in the distribution of species in fragmented habitat. *Oecologia*, 96(3):373–382, 1993.

Stefan Milton Bache and Hadley Wickham. *magrittr: A Forward-Pipe Operator for R*, 2014. URL `https://CRAN.R-project.org/package=magrittr`. R package version 1.5.

John W Baird. The selection and use of fruit by birds in an eastern forest. *The Wilson Bulletin*, pages 63–73, 1980.

Ralph V Bangham. Studies on fish parasites of lake huron and manitoulin island. *American Midland Naturalist*, pages 184–194, 1955.

Pedro Barbosa, Astrid Caldas, and Gaden Robinson. Host plant associations among species in two macrolepidopteran assemblages. *Journal of Entomological Science*, 38(1):41–47, 2003.

Rodolphe Barrangou, Sung-Sik Yoon, Frederick Breidt Jr, Henry P Fleming, and Todd R Klaenhammer. Characterization of six leuconostoc fallax bacteriophages isolated from an industrial sauerkraut fermentation. *Applied and environmental microbiology*, 68(11):5452–5458, 2002.

Spencer CH Barrett and Kaius Helenurm. The reproductive biology of boreal forest herbs. i. breeding systems and pollination. *Canadian Journal of Botany*, 65(10):2036–2046, 1987.

Ignasi Bartomeus, Montserrat Vilà, and Luís Santamaría. Contrasting effects of invasive plants in plant–pollinator networks. *Oecologia*, 155(4):761–770, 2008.

Yves Basset, GA Samuelson, and SE Miller. Similarities and contrasts in the local insect faunas associated with ten forest tree species of new guinea1. *Pacific Science*, 50(2):157–183, 1996.

Bruce Beehler. Frugivory and polygamy in birds of paradise. *The Auk*, pages 1–12, 1983.

Sybelle Bellay, Edson Fontes de Oliveira, Mário Almeida-Neto, Dilermando Pereira Lima Junior, Ricardo Massato Takemoto, and José Luis Luque. Developmental stage of parasites influences the structure of fish-parasite networks. *PloS one*, 8(10):e75710, 2013.

Elisângela LS Bezerra, Isabel C Machado, and Marco AR Mello. Pollination networks of oil-flowers: A tiny world within the smallest of all worlds. *Journal of Animal Ecology*, 78(5):1096–1101, 2009.

Nico Blüthgen, Nigel E Stork, and Konrad Fiedler. Bottom-up control and co-occurrence in complex communities: honeydew and nectar determine a rainforest ant mosaic. *Oikos*, 106(2):344–358, 2004.

Nico Blüthgen, Florian Menzel, and Nils Blüthgen. Measuring specialization in species interaction networks. *BMC ecology*, 6(1):9, 2006.

Melissa R Bodner. *The biogeography and age of salticid spider radiations with the introduction of a new African group (Araneae: Salticidae).* PhD thesis, University of British Columbia, 2009.

Berry J Brosi, Kyle Niezgoda, and Heather M Briggs. Experimental species removals impact the architecture of pollination networks. *Biology letters*, 13(6):20170243, 2017a.

BJ Brosi, K Niezgoda, and HM Briggs. Data from: Experimental species removals impact the architecture of pollination networks, 2017b. URL `https://doi.org/10.5061/dryad.b5h65`.

M Bundgaard. *Tidslig og rumlig variation i et plante-bestøvernetværk*. PhD thesis, MSc. thesis, Aarhus University, Aarhus. FROM BROADSTONE TO ZACKENBERG 57, 2003.

Laura A Burkle, John C Marlin, and Tiffany M Knight. Plant-pollinator interactions over 120 years: loss of species, co-occurrence, and function. *Science*, 339(6127):1611–1615, 2013.

Rosanna Capparelli, Nunzia Nocerino, Marco Iannaccone, Danilo Ercolini, Marianna Parlato, Medaglia Chiara, and Domenico Iannelli. Bacteriophage therapy of salmonella enterica: a fresh appraisal of bacteriophage therapy. *Journal of Infectious Diseases*, 201(1):52–61, 2010.

Tomás A Carlo, Jaime A Collazo, and Martha J Groom. Avian fruit preferences across a puerto rican forested landscape: pattern consistency and implications for seed removal. *Oecologia*, 134(1):119–131, 2003.

Daniel W Carstensen, Kristian Trøjelsgaard, Jeff Ollerton, and Leonor Patricia C Morellato. Local and regional specialization in plant–pollinator networks. *Oikos*, 127(4):531–537, 2018.

DW Carstensen, K Trøjelsgaard, J Ollerton, and LPC Moraletto. Data from: Local and regional specialization in plant–pollinator networks, 2017. URL `https://doi.org/10.5061/dryad.c45cd`.

LG Carvalheiro, ERM Barbosa, and J Memmott. Pollinator networks, alien species and the conservation of rare plants: Trinia glauca as a case study. *Journal of Applied Ecology*, 45(5):1419–1427, 2008.

Fan Chung, Linyuan Lu, and Van Vu. Spectra of random graphs with given expected degrees. *Proceedings of the National Academy of Sciences*, 100(11):6313–6318, 2003.

Frederic Edward Clements and Frances Louise Long. *Experimental pollination: an outline of the ecology of flowers and insects*. Carnegie Institution of Washington, 1923.

PD Coley, ML Bateman, and TA Kursar. The effects of plant quality on caterpillar growth and defense against natural enemies. *Oikos*, 115(2):219–228, 2006.

André M Comeau, Enrico Buenaventura, and Curtis A Suttle. A persistent, productive, and seasonally dynamic vibriophage population within pacific oysters (crassostrea gigas). *Applied and environmental microbiology*, 71(9):5324–5331, 2005.

André M Comeau, Amy M Chan, and Curtis A Suttle. Genetic richness of vibriophages isolated in a coastal environment. *Environmental microbiology*, 8(7):1164–1176, 2006.

FHJ Crome. The ecology of fruit pigeons in tropical northern queensland. *Wildlife Research*, 2(2):155–185, 1975.

Gabor Csardi and Tamas Nepusz. The igraph software package for complex network research. *InterJournal*, Complex Systems:1695, 2006. URL `http://igraph.org`.

David B. Dahl. *xtable: Export Tables to LaTeX or HTML*, 2016. URL `https://CRAN.R-project.org/package=xtable`. R package version 1.8-2.

Diane W Davidson, Roy R Snelling, and John T Longino. Competition among ants for myrmecophytes and the significance of plant trichomes. *Biotropica*, pages 64–73, 1989.

Gilberto M de Mendonça Santos, Cândida M Lima Aguiar, and Marco AR Mello. Flower-visiting guild associated with the caatinga flora: trophic interaction networks formed by social bees and social wasps with plants. *Apidologie*, 41(4):466–475, 2010.

Alex O Dechtiar. Parasites of fish from lake of the woods, ontario. *Journal of the Fisheries Board of Canada*, 29(3):275–283, 1972.

LV Dicks, SA Corbet, and RF Pywell. Compartmentalization in plant–insect flower visitor webs. *Journal of Animal Ecology*, 71(1):32–43, 2002.

Katsumi Doi, Ye Zhang, Yousuke Nishizaki, Akiko Umeda, Sadahiro Ohmomo, and Seiya Ogata. A comparative study and phage typing of silage-making lactobacillus bacteriophages. *Journal of Bioscience and Bioengineering*, 95(5):518–525, 2003.

C. F. Dormann. How to be a specialist? quantifying specialisation in pollination networks. *Network Biology*, 1(1):1–20, 2011.

C. F. Dormann, B. Gruber, and J. Fruend. Introducing the bipartite package: Analysing ecological networks. *R News*, 8(2):8–11, 2008.

C. F. Dormann, J. Frueund, N. Bluethgen, and B. Gruber. Indices, graphs and null models: analyzing bipartite ecological networks. *The Open Ecology Journal*, 2:7–24, 2009.

Yoko L Dupont and Jens M Olesen. Ecological modules and roles of species in heathland plant–insect flower visitor networks. *Journal of Animal Ecology*, 78(2):346–353, 2009.

Yoko L Dupont, Dennis M Hansen, and Jens M Olesen. Structure of a plant–flower-visitor network in the high-altitude sub-alpine desert of tenerife, canary islands. *Ecography*, 26(3):301–310, 2003.

Heidi Elberling and Jens M Olesen. The structure of a high latitude plant-flower visitor system: the dominance of flies. *Ecography*, 22(3):314–323, 1999.

Miroslav Fiedler. Algebraic connectivity of graphs. *Czechoslovak mathematical journal*, 23(2):298–305, 1973.

Cesar O Flores, Justin R Meyer, Sergi Valverde, Lauren Farr, and Joshua S Weitz. Statistical structure of host–phage interactions. *Proceedings of the National Academy of Sciences*, 108(28):E288–E297, 2011.

Carlos Roberto Fonseca and Gislene Ganade. Asymmetries, compartments and null interactions in an amazonian ant-plant community. *Journal of Animal Ecology*, pages 339–347, 1996.

PGH Frost. Fruit-frugivore interactions in a south african coastal dune forest, 1980.

M Galletti and MA Pizo. Fruit cating birds in a forest fragment in southcastern brazil. *Ararajuba*, 4:71–79, 1996.

L Gilarranz, M Sabatino, M Aizen, and J Bascompte. Data from: Hotspots of mutualistic networks, 2014. URL `https://doi.org/10.5061/dryad.cr3ft`.

Luis J Gilarranz, Malena Sabatino, Marcelo A Aizen, and Jordi Bascompte. Hot spots of mutualistic networks. *Journal of Animal Ecology*, 84(2):407–413, 2015.

J Guitian Rivera and A Callejo Rey. Structure d'une communauté de carnivores dans la cordillère cantabrique occidentale. *Revue d'écologie*, 37(2):145–160, 1983.

Jarrod D Hadfield, Boris R Krasnov, Robert Poulin, and Shinichi Nakagawa. A tale of two phylogenies: comparative analyses of ecological interactions. *The American Naturalist*, 183(2):174–187, 2013.

Andreas Hamann and Eberhard Curio. Interactions among frugivores and fleshy fruit trees in a philippine submontane rainforest. *Conservation Biology*, 13(4):766–773, 1999.

Vinni M Hansen, Hanne Rosenquist, Dorte L Baggesen, Stanley Brown, and Bjarke B Christensen. Characterization of campylobacter phages including analysis of host range by selected campylobacter penner serotypes. *BMC microbiology*, 7(1):90, 2007.

Javier Herrera. Pollination relationships in southern spanish mediterranean shrublands. *The Journal of Ecology*, pages 274–287, 1988.

Brian Hocking. Insect-flower associations in the high arctic with special reference to nectar. *Oikos*, pages 359–387, 1968.

Karin Holmfeldt, Mathias Middelboe, Ole Nybroe, and Lasse Riemann. Large variabilities in host strain susceptibility and phage host range govern interactions between lytic marine phages and their flavobacterium hosts. *Applied and environmental Microbiology*, 73(21):6730–6739, 2007.

Sebastien Ibanez, Sandra Lavorel, Sara Puijalon, and Marco Moretti. Herbivory mediated by coupling between biomechanical traits of plants and grasshoppers. *Functional Ecology*, 27(2):479–489, 2013.

Ken Inoue. Evolution of mating systems in island populations of campanula microdonta: pollinator availability hypothesis. *Plant species biology*, 5(1):57–64, 1990.

David W Inouye and Graham H Pyke. Pollination biology in the snowy mountains of australia: comparisons with montane colorado, usa. *Australian Journal of Ecology*, 13(2):191–205, 1988.

Daniel H Janzen, ST Doerner, and Eric E Conn. Seasonal constancy of intra-population variation of hcn content of costa rican acacia farnesiana foliage. *Phytochemistry*, 19(9):2022–2023, 1980.

Daniel H Janzen, AK Walker, James B Whitfield, Gérard Delvare, and Ian D Gauld. Host-specificity and hyperparasitoids of three new costa rican species of microplitis foerster (hymenoptera: Braconidae: Microgastrinae), parasitoids of sphingid caterpillars. *Journal of Hymenoptera Research*, 12(1):42–76, 2003.

Anthony Joern. Feeding patterns in grasshoppers (orthoptera: Acrididae): factors influencing diet specialization. *Oecologia*, 38(3):325–347, 1979.

Pedro Jordano. El ciclo anual de los paseriformes frugívoros en el matorral mediterráneo del sur de españa: importancia de su invernada y variaciones interanuales. *Ardeola*, 32(1):69–94, 1985.

Christopher N Kaiser-Bunbury, Stefanie Muff, Jane Memmott, Christine B Müller, and Amedeo Caflisch. The robustness of pollination networks to the loss of species and interactions: a quantitative approach incorporating pollinator behaviour. *Ecology letters*, 13(4):442–452, 2010.

Christopher N Kaiser-Bunbury, Diego P Vázquez, Martina Stang, and Jaboury Ghazoul. Determinants of the microstructure of plant–pollinator networks. *Ecology*, 95(12):3314–3324, 2014.

Takehiko Kakutani, Tamiji Inoue, Makoto Kato, and Hideyuki Ichihashi. Insect-flower relationship in the campus of kyoto university, kyoto: an overview of the flowering phenology and the seasonal pattern of insect visits. *Contribution from Biological Laboratory, Kyoto University*, 27:465–521, 1990.

Jyri Kankila and Kristina Lindström. Host range, morphology and dna restriction patterns of bacteriophage isolates infecting *Rhizobium leguminosarum* bv. *trifolii*. *Soil Biology and Biochemistry*, 26(4):429–437, 1994.

M Kato. Anthophilous insect community and plant-pollinator interactions on amami islands in the ryukyo archipelago, japan. *Contribution from Biological Laboratory, Kyoto University*, 29:157–252, 2000.

M Kato, M Matsumoto, and T Kato. Flowering phenology and anthophilous insect community in the cool-temperate subalpine forests and meadows at mt. kushigata in the central part of japan. *Contribution from Biological Laboratory, Kyoto University*, 28:119–172, 1993.

Makoto Kato. Plant-pollinator interactions in the understory of a lowland mixed dipterocarp forest in sarawak. *American Journal of Botany*, pages 732–743, 1996.

Makoto Kato, Takehiko Kakutani, Tamiji Inoue, Takao Itino, et al. Insect-flower relationship in the primary beech forest of ashu, kyoto: an overview of the flowering phenology and the seasonal pattern of insect visits. 1990.

PG Kevan. High arctic insect-flower visitor relations: the inter-relationships of arthropods and flowers at lake hazen, ellesmere island, northwest territories, canada. *Univ. of Alberta, Canada*, 1970. Ph.D. Thesis.

Boris R Krasnov, Miguel A Fortuna, David Mouillot, Irina S Khokhlova, Georgy I Shenbrot, and Robert Poulin. Phylogenetic signal in module composition and species connectivity in compartmentalized host-parasite networks. *The American Naturalist*, 179(4):501–511, 2012.

VN Krylov, S Miller, R Rachel, M Biebl, EA Pleteneva, M Schuetz, SV Krylov, and OV Shaburova. Ambivalent bacteriophages of different species active on escherichia coli k12 and salmonella sp. strains. *Russian Journal of Genetics*, 42(2):106–114, 2006.

Frank Lambert. Fig-eating by birds in a malaysian lowland rain forest. *Journal of Tropical Ecology*, 5(4): 401–412, 1989.

Ross Langley, Dervla T Kenna, Peter Vandamme, Rebecca Ure, and John RW Govan. Lysogeny and bacteriophage host range within the burkholderia cepacia complex. *Journal of medical microbiology*, 52 (6):483–490, 2003.

C Lara-Romero, C García, J Morente-López, and JM Iriondo. Data from: Direct and indirect effects of shrub encroachment on alpine grasslands mediated by plant-flower visitor interactions, 2016a. URL `https://doi.org/10.5061/dryad.p869n`.

Carlos Lara-Romero, Cristina García, Javier Morente-López, and José M Iriondo. Direct and indirect effects of shrub encroachment on alpine grasslands mediated by plant–flower visitor interactions. *Functional Ecology*, 30(9):1521–1530, 2016b.

J Le Corff, RJ Marquis, and JB Whitfield. Temporal and spatial variation in a parasitoid community associated with the herbivores that feed on missouri quercus. *Environmental Entomology*, 29(2):181–194, 2000.

Simon R Leather. Feeding specialisation and host distribution of british and finnish prunus feeding macrolepidoptera. *Oikos*, 60(1):40–48, 1991.

TS Leong and JC Holmest. Communities of metazoan parasites in open water fishes of cold lake, alberta. *Journal of Fish Biology*, 18(6):693–713, 1981.

Owen T Lewis, Jane Memmott, John Lasalle, Chris HC Lyal, Caroline Whitefoord, and H Charles J Godfray. Structure of a diverse tropical forest insect–parasitoid community. *Journal of Animal Ecology*, 71(5):855–873, 2002.

Andy Liaw and Matthew Wiener. Classification and regression by randomforest. *R News*, 2(3):18–22, 2002. URL `https://CRAN.R-project.org/doc/Rnews/`.

Pedro G Lind, Marta C Gonzalez, and Hans J Herrmann. Cycles and clustering in bipartite networks. *Physical review E*, 72(5):056127, 2005.

J Loye and M Zuk. Ecology, evolution and behaviour in avian-parasite interactions, 1992.

Rebekka Lundgren and Jens M Olesen. The dense and highly connected world of greenland's plants and their pollinators. *Arctic, Antarctic, and Alpine Research*, 37(4):514–520, 2005.

Sarina Macfadyen, Rachel Gibson, Andrew Polaszek, Rebecca J Morris, Paul G Craze, Robert Planqué, William OC Symondson, and Jane Memmott. Do differences in food web structure between organic and conventional farms affect the ecosystem service of pest control? *Ecology Letters*, 12(3):229–238, 2009.

Andrew L Mack and Debra D Wright. Notes on occurrence and feeding of birds at crater mountain biological research station, papua new guinea. *Emu*, 96(2):89–101, 1996.

A Magrach, A Holzschuh, I Bartomeus, V Riedinger, SPM Roberts, M Rundlöf, A Vujic, JB Wickens, VJ Wickens, R Bommarco, JP Gonzalez-Varo, SG Potts, HG Smith, I Steffan-Dewenter, and M Vilà. Data from: Plant-pollinator networks in semi-natural grasslands are resistant to the loss of pollinators during blooming of mass-flowering crops, 2017. URL `https://doi.org/10.5061/dryad.k0q1n`.

Ainhoa Magrach, Andrea Holzschuh, Ignasi Bartomeus, Verena Riedinger, Stuart PM Roberts, Maj Rundlöf, Ante Vujić, Jennifer B Wickens, Victoria J Wickens, Riccardo Bommarco, et al. Plant–pollinator networks in semi-natural grasslands are resistant to the loss of pollinators during blooming of mass-flowering crops. *Ecography*, 41(1):62–74, 2018.

Vladimir Alexandrovich Marchenko and Leonid Andreevich Pastur. Distribution of eigenvalues for some sets of random matrices. *Matematicheskii Sbornik*, 114(4):507–536, 1967.

CK McMullen. Flower-visiting insects of the galapagos islands. *Pan-Pacific Entomologist*, 69(1):95–106, 1993.

Diego Medan, Norberto H Montaldo, Mariano Devoto, Anita Mantese, Viviana Vasellati, Germán G Roitman, and Norberto H Bartoloni. Plant-pollinator relationships at two altitudes in the andes of mendoza, argentina. *Arctic, Antarctic, and Alpine Research*, pages 233–241, 2002.

Jane Memmott. The structure of a plant-pollinator food web. *Ecology Letters*, 2(5):276–280, 1999.

Mathias Middelboe, Karin Holmfeldt, Lasse Riemann, Ole Nybroe, and Jakob Haaber. Bacteriophages drive strain diversification in a marine flavobacterium: implications for phage resistance and physiological properties. *Environmental microbiology*, 11(8):1971–1982, 2009.

Andreja Miklič and Irena Rogelj. Characterization of lactococcal bacteriophages isolated from slovenian dairies. *International journal of food science & technology*, 38(3):305–311, 2003.

A Montero. *The ecology of three pollination networks.* PhD thesis, MSc thesis, Aarhus University, Aarhus, 2005.

T Mosquin and J Martin. Observations on the pollination biology of plants on melville island, nwt, canada. *Canadian Field Naturalist*, 81:201–205, 1967.

Alexander F Motten. *Pollination Ecology of the Spring Wildflower Community in the Deciduous Forests of Piedmont North Carolina.* PhD thesis, Duke University, Duhram, North Carolina, USA, 1982.

Shinichi Nakagawa, Robert Poulin, Kerrie Mengersen, Klaus Reinhold, Leif Engqvist, Malgorzata Lagisz, and Alistair M Senior. Meta-analysis of variation: ecological and evolutionary applications and beyond. *Methods in Ecology and Evolution*, 6(2):143–152, 2015.

Mark EJ Newman. Modularity and community structure in networks. *Proceedings of the national academy of sciences*, 103(23):8577–8582, 2006.

Naohiko NOMA. Annual fluctuations of sapfruits production and synchronization within and inter species in a warm temperate forest on yakushima island. *Tropics*, 6:441–449, 1997.

Vojtech Novotny, Scott E Miller, Yves Basset, Lukas Cizek, Karolyn Darrow, Borenke Kaupa, Joseph Kua, and George D Weiblen. An altitudinal comparison of caterpillar (lepidoptera) assemblages on ficus trees in papua new guinea. *Journal of Biogeography*, 32(8):1303–1314, 2005.

Vojtech Novotny, Scott E Miller, Jan Hrcek, Leontine Baje, Yves Basset, Owen T Lewis, Alan JA Stewart, and George D Weiblen. Insects on plants: explaining the paradox of low diversity within specialist herbivore guilds. *The American Naturalist*, 179(3):351–362, 2012.

Jens M Olesen, Louise I Eskildsen, and Shadila Venkatasamy. Invasion of pollination networks on oceanic islands: importance of invader complexes and endemic super generalists. *Diversity and Distributions*, 8 (3):181–192, 2002.

JEFF Ollerton, Steven D Johnson, LOUISE CRANMER, and SAM Kellie. The pollination ecology of an assemblage of grassland asclepiads in south africa. *Annals of Botany*, 92(6):807–834, 2003.

Jeff Ollerton, Duncan McCollin, Daphne G Fautin, and Gerald R Allen. Finding nemo: nestedness engendered by mutualistic organization in anemonefish and their hosts. *Proceedings of the Royal Society B: Biological Sciences*, 274(1609):591–598, 2007.

Tore Opsahl. Triadic closure in two-mode networks: Redefining the global and local clustering coefficients. *Social Networks*, 35(2):159–167, 2013.

KA Orford, J Memmott, IP Vaughan, and PJ Murray. Data from: Modest enhancements to conventional grassland diversity improve the provision of pollination services, 2016a. URL `https://doi.org/10.5061/dryad.tp0d0`.

Katherine A Orford, Phil J Murray, Ian P Vaughan, and Jane Memmott. Modest enhancements to conventional grassland diversity improve the provision of pollination services. *Journal of Applied Ecology*, 53(3): 906–915, 2016b.

Heather A Passmore, Emilio M Bruna, Sylvia M Heredia, and Heraldo L Vasconcelos. Resilient networks of ant-plant mutualists in amazonian forest fragments. *PloS one*, 7(8):e40803, 2012.

Romualdo Pastor-Satorras and Claudio Castellano. Distinct types of eigenvector localization in networks. *Scientific reports*, 6:18847, 2016.

Mary Percival. Floral ecology of coastal scrub in southeast jamaica. *Biotropica*, pages 104–129, 1974.

Th Petanidou. Pollinating fauna of a phryganic ecosystem: species list. *Verslagen en Technische Gegevens*, 59(1):1–11, 1991.

Shai Pilosof, Miguel A Fortuna, Maxim V Vinarski, Natalia P Korallo-Vinarskaya, and Boris R Krasnov. Temporal dynamics of direct reciprocal and indirect effects in a host–parasite network. *Journal of Animal Ecology*, 2013.

Brigitte Poulin, S Joseph Wright, Gaetan Lefebvre, and Osvaldo Calderon. Interspecific synchrony and asynchrony in the fruiting phenologies of congeneric bird-dispersed plants in panama. *Journal of Tropical Ecology*, 15(2):213–227, 1999.

Virginie Poullain, Sylvain Gandon, Michael A Brockhurst, Angus Buckling, and Michael E Hochberg. The evolution of specificity in evolving and coevolving antagonistic interactions between a bacteria and its phage. *Evolution*, 62(1):1–11, 2008.

Paulo Inácio Prado and Thomas Michael Lewinsohn. Compartments in insect–plant associations and their consequences for community structure. *Journal of Animal Ecology*, 73(6):1168–1178, 2004.

Richard B Primack. Insect pollination in the new zealand mountain flora. *New Zealand Journal of Botany*, 21(3):317–333, 1983.

A Quiberoni, L Auad, AG Binetti, VB Suárez, JA Reinheimer, and RR Raya. Comparative analysis of *Streptococcus thermophilus* bacteriophages isolated from a yogurt industrial plant. *Food microbiology*, 20 (4):461–469, 2003.

R Core Team. *R: A Language and Environment for Statistical Computing*. R Foundation for Statistical Computing, Vienna, Austria, 2018. URL `https://www.R-project.org/`.

Nelson Ramirez. Biología de polinización en una comunidad arbustiva tropical de la alta guayana venezolana. *Biotropica*, pages 319–330, 1989.

Nelson Ramirez and Ysaleny Brito. Pollination biology in a palm swamp community in the venezuelan central plains. *Botanical Journal of the Linnean Society*, 110(4):277–302, 1992.

Charles Robertson. *Flowers and Insects: lists of visitors of four hundred and fifty three flowers*. The Science Press Printing Company, Carlinville, IL, 1929.

David Robinson. *broom: Convert Statistical Analysis Objects into Tidy Data Frames*, 2017. URL `https://CRAN.R-project.org/package=broom`. R package version 0.4.2.

Jan Rybniker, Stefanie Kramme, and Pamela L Small. Host range of 14 mycobacteriophages in mycobacterium ulcerans and seven other mycobacteria including mycobacterium tuberculosis–application for identification and susceptibility testing. *Journal of medical microbiology*, 55(1):37–42, 2006.

Douglas W Schemske, Mary F Willson, Michael N Melampy, Linda J Miller, Louis Verner, Kathleen M Schemske, and Louis B Best. Flowering ecology of some spring woodland herbs. *Ecology*, pages 351–366, 1978.

Matthias Schleuning, Nico Blüthgen, Martina Flörchinger, Julius Braun, H Martin Schaefer, and Katrin Böhning-Gaese. Specialization and interaction strength in a tropical plant-frugivore network differ among forest strata. *Ecology*, 92(1):26–36, 2011.

Kimberley D Seed and Jonathan J Dennis. Isolation and characterization of bacteriophages of the burkholderia cepacia complex. *FEMS microbiology letters*, 251(2):273–280, 2005.

WR Silva. Patterns of fruit-frugivore interactions in two atlantic forest bird communities of south-eastern brazil: implications for conservation. *Seed dispersal and frugivory: ecology, evolution and conservation*, pages 423–435, 2002.

Ernst Small. Insect pollinators of the mer bleue peat bog of ottawa. *Canadian Field Naturalist*, 90:22–28, 1976.

Cecilia Smith-Ramírez, P Martinez, M Nunez, C González, and Juan J Armesto. Diversity, flower visitation frequency and generalism of pollinators in temperate rain forests of chiloé island, chile. *Botanical Journal of the Linnean Society*, 147(4):399–416, 2005.

B Snow and D Snow. Birds and berries–a study of ecological interactions. *Poyser, London*, 1988.

Barbara K Snow and DW Snow. The feeding ecology of tanagers and honeycreepers in trinidad. *The Auk*, 88(2):291–322, 1971.

AE Sorensen. Interactions between birds and fruit in a temperate woodland. *Oecologia*, 50(2):242–249, 1981.

L Stald. *Pollination Networks on the Canary Islands*. PhD thesis, MSc thesis, Aarhus University, Aarhus, 2003.

Phillip PA Staniczenko, Jason C Kopp, and Stefano Allesina. The ghost of nestedness in ecological networks. *Nature communications*, 4:1391, 2013.

Petr Starỳ, Jan Havelka, et al. Fauna and associations of aphid parasitoids in an up-dated farmland area (czech republic). *Bulletin of Insectology*, 61(2):251–276, 2008.

Anne Rønnest Stenholm, Inger Dalsgaard, and Mathias Middelboe. Isolation and characterization of bacteriophages infecting the fish pathogen flavobacterium psychrophilum. *Applied and environmental microbiology*, 74(13):4070–4078, 2008.

John O Stireman and Michael S Singer. Determinants of parasitoid–host associations: insights from a natural tachinid–lepidopteran community. *Ecology*, 84(2):296–310, 2003.

Giovanni Strona and Joseph A Veech. A new measure of ecological network structure based on node overlap and segregation. *Methods in Ecology and Evolution*, 6(8):907–915, 2015.

Giovanni Strona, Domenico Nappo, Francesco Boccacci, Simone Fattorini, and Jesus San-Miguel-Ayanz. A fast and unbiased procedure to randomize ecological binary matrices with fixed row and column totals. *Nature communications*, 5:4114, 2014.

Shinji Sugiura. Structure of a herbivore–parasitoid community: Are parasitoids shared by different herbivore guilds? *Basic and Applied Ecology*, 8(6):544–551, 2007.

Matthew B Sullivan, John B Waterbury, and Sallie W Chisholm. Cyanophages infecting the oceanic cyanobacterium prochlorococcus. *Nature*, 424(6952):1047–1051, 2003.

Aidan J Synnott, Ying Kuang, Miki Kurimoto, Keiko Yamamichi, Hidetomo Iwano, and Yasunori Tanji. Isolation from sewage influent and characterization of novel staphylococcus aureus bacteriophages with wide host ranges and potent lytic capabilities. *Applied and environmental microbiology*, 75(13):4483–4490, 2009.

Gérard Tavakilian, Amy Berkov, Barbara Meurer-Grimes, and Scott Mori. Neotropical tree species and their faunas of xylophagous longicorns (coleoptera: Cerambycidae) in french guiana. *The botanical review*, 63 (4):303–355, 1997.

Elisa Thébault and Colin Fontaine. Stability of ecological communities and the architecture of mutualistic and trophic networks. *Science*, 329(5993):853–856, 2010.

Kristian Trøjelsgaard, Pedro Jordano, Daniel W Carstensen, and Jens M Olesen. Geographical variation in mutualistic networks: similarity, turnover and partner fidelity. *Proc. R. Soc. B*, 282(1802):20142925, 2015.

K Trøjelsgaard, P Jordano, DW Carstensen, and JM Olesen. Data from: Geographical variation in mutualistic networks: similarity, turnover and partner fidelity, 2015. URL `https://doi.org/10.5061/dryad.76173`.

Cristina Tur, Beatriz Vigalondo, Kristian Trøjelsgaard, Jens M Olesen, and Anna Traveset. Downscaling pollen–transport networks to the level of individuals. *Journal of Animal Ecology*, 2013.

Caroline EG Tutin, Rebecca M Ham, Lee JT White, and Michael JS Harrison. The primate community of the lopé reserve, gabon: diets, responses to fruit scarcity, and effects on biomass. *American journal of primatology*, 42(1):1–24, 1997.

Darrell N Ueckert, Michael C Bodine, and Brian M Spears. Population density and biomass of the desert termite gnathamitermes tubiformans (isoptera: Termitidae) in a shortgrass prairie: relationship to temperature and moisture. *Ecology*, 57(6):1273–1280, 1976.

Diego P Vázquez. *Interactions among introduced ungulates, plants, and pollinators: a field study in the temperate forest of the southern Andes*. PhD thesis, University of Tennessee, Knoxville, 2002.

Christiane Natalie Weiner, Michael Werner, Karl Eduard Linsenmair, and Nico Blüthgen. Land use intensity in grasslands: Changes in biodiversity, species composition and specialisation in flower visitor networks. *Basic and Applied Ecology*, 12(4):292–299, 2011.

Bernard L Welch. The generalization ofstudent's' problem when several different population variances are involved. *Biometrika*, 34(1/2):28–35, 1947.

Nathaniel T Wheelwright, William A Haber, K Greg Murray, and Carlos Guindon. Tropical fruit-eating birds and their food plants: a survey of a costa rican lower montane forest. *Biotropica*, pages 173–192, 1984.

Antje Wichels, Stefan S Biel, Hans R Gelderblom, Thorsten Brinkhoff, Gerard Muyzer, and Christian Schütt. Bacteriophage diversity in the north sea. *Applied and environmental microbiology*, 64(11):4128–4133, 1998.

Hadley Wickham. *scales: Scale Functions for Visualization*, 2017a. URL `https://CRAN.R-project.org/package=scales`. R package version 0.5.0.

Hadley Wickham. *stringr: Simple, Consistent Wrappers for Common String Operations*, 2017b. URL `https://CRAN.R-project.org/package=stringr`. R package version 1.2.0.

Hadley Wickham. *tidyverse: Easily Install and Load 'Tidyverse' Packages*, 2017c. URL `https://CRAN.R-project.org/package=tidyverse`. R package version 1.1.1.

K Yamazaki and M Kato. Flowering phenology and anthophilous insect community in a grassland ecosystem at mt. yufu, western japan. *Contribution from Biological Laboratory, Kyoto University*, 29:255–318, 2003.

Peng Zhang, Jinliang Wang, Xiaojia Li, Menghui Li, Zengru Di, and Ying Fan. Clustering coefficient and community structure of bipartite networks. *Physica A: Statistical Mechanics and its Applications*, 387 (27):6869–6875, 2008.

P Zinno, T Janzen, M Bennedsen, D Ercolini, and G Mauriello. Characterization of *Streptococcus thermophilus* lytic bacteriophages from mozzarella cheese plants. *International journal of food microbiology*, 138(1):137–144, 2010.
